# Antirheumatoid Arthritic Effects of *Sabia parviflora* Wall. Leaf Extracts *via* the NF-κB Pathway and Transient Receptor Potential Protein Family

**DOI:** 10.3389/fphar.2022.880350

**Published:** 2022-06-16

**Authors:** Yongqiang Zhou, Yamin Zhao, Hui Xu, Xiaoyan Zhao, Chunli Zhao, Tao Zhou, Yongping Zhang

**Affiliations:** ^1^ College of Pharmacy, Guizhou University of Traditional Chinese Medicine, Guiyang, China; ^2^ Resource Institute for Chinese & Ethnic Materia Medica, Gui Zhou University of Traditional Chinese Medicine, Guiyang, China

**Keywords:** *Sabia parviflora* Wall., TRP family, NF-κB, rheumatoid arthritis, flavonoids

## Abstract

As an important traditional medicine of Buyi and Miao ethnic groups in Guizhou, *Sabia parviflora* Wall. provides antiviral properties against hepatitis, eliminates wind and dampness, and exhibits anti-inflammatory and pain relief properties. It has also been shown to treat rheumatoid arthritis (RA) and other diseases. However, the pharmacodynamic mechanism of *S. parviflora* Wall. for RA has not been reported. In this study, we identified the effective compounds of *S. parviflora* Wall*.* leaves against RA and discussed the mechanism against complete Freund’s adjuvant-induced arthritis (AIA) based on inflammatory proteins and transient receptor potential (TRP) proteins. *S. parviflora* Wall. leaf extracts (0.64 g/kg, 0.32 g/kg, and 0.16 g/kg, once daily) were given orally for 21 days. On the 15th day of complete Freund’s adjuvant-induced RA, the effects of this medicine on RA rats were investigated. *S. parviflora* Wall. extracts increased body weight, decreased foot swelling, and reduced thymus and spleen indices in model rats. Most of pannus in the synovial tissue of RA rats disappeared upon treatment, and the local inflammatory cells were greatly reduced when given the fraction of n-butanol (0.64 g/kg/d, 0.32 g/kg/d, and 0.16 g/kg/d) of 70% alcohol-soluble fraction of *S. parviflora* Wall. leaves. In addition, the release of inflammatory factors such as tumor necrosis factor-α (TNF-α), interleukin-1β (IL-1β), interleukin-6 (IL-6), interleukin-10 (IL-10), interleukin-15 (IL-15), and vascular endothelial growth factor (VEGF) in the RA rat serum was inhibited. The active compounds inhibited the expression of TNF-α, IL-1β, IL-6, IL-10, IL-15 and nuclear factor kappa-Bp65 (NF-κBp65) inflammatory protein and TRP protein transient receptor potential melastatin-5 (TRPM-5) and transient receptor potential channel-6 (TRPC-6), to reduce the expression of VEGF in synovial tissue of RA rats and relieve redness and edema. High-performance liquid chromatography identified six flavonoids and three triterpenoid saponins as active compounds. These findings suggest *S. parviflora* Wall. leaves may play a role in RA treatment by inhibiting the release of inflammatory factors as well as participating in the inflammatory protein expression in the NF-κB pathway and TRP protein family.

## Introduction

Rheumatoid arthritis (RA) is a chronic, systemic autoimmune disease with the proliferation of synovial tissue, infiltration of inflammatory cells, and pannus formation. It gradually leads to the irreversible damage of cartilage and bone, resulting in different degrees of the loss of joint function in RA patients (Aletaha et al., 2018; Li et al., 2019). Currently, the exact pathogenesis of RA remains unclear. Therefore, research on the etiology and pathological mechanism of RA and the development of therapeutic drugs are imperative. Numerous studies have shown that synovial cells, monocytes/macrophages, and lymphocytes can produce a large number of inflammatory cytokines when RA occurs, which have important internal relationships with the emergence and development of the disease (Falconer et al., 2018; Pala et al., 2018; Siouti et al., 2019; Saleem et al., 2020). At present, the drugs used to treat RA on the market are mainly non-steroidal anti-inflammatory drugs, adrenal glucocorticoids, and biological agents. Although these drugs can relieve pain and reduce inflammatory response, they are prone to serious side effects, such as teratogenicity, osteoporosis, and osteonecrosis. (Chatzidionysiou et al., 2017; Schultz et al., 2017). In contrast, Chinese herbal medicine has attracted extensive attention because of its unique curative effects, low toxicity, minimal side effects, and great development potential. It plays a very important role in the treatment of RA and has obvious characteristics and advantages.

*Sabia parviflora* Wall. is a deciduous woody vine belonging to the genus *Sabia* of Sabiaceae with small yellow flowers and red fruits. It is also known as “xiaohuaqingfengteng” in Chinese. Its leaves are considered a vital Miao medicine, and it has been widely used to treat RA by Buyi and Miao ethnic groups in southwest Guizhou Province with no scientific validation (Sui et al., 2011; Chen et al., 2020). The crude extract of *S. parviflora* Wall. has pharmacological activities against acute liver injury and influenza virus (Chen et al., 2015; Zhao et al., 2018). At present, the research on the chemical constituents of *S. parviflora* Wall. mainly focuses on triterpenoids, alkaloids, flavonoids, and other compounds (Fan et al., 2018; Cui et al., 2022; Zhou et al., 2022). However, the pharmacodynamic mechanism of *S. parviflora* Wall. leaves for the treatment of RA has not been reported.

In this research, we identified the bioactive compounds of *S. parviflora* Wall. leaf extracts for the treatment of RA in a rat model. We also measured the inflammatory cytokine levels in the serum of RA rats and the pathogenesis-related protein expression in synovial tissue in order to elucidate the possible mechanism.

## Materials and Methods

### Chemicals and Reagents

Complete Freund’s adjuvant (CFA) was obtained from Sigma Chemical Co. (St.Louis, MO, United States), and dexamethasone was purchased from Hubei Tianyao Pharmaceutical Co. (Wuhan, China). Tumor necrosis factor-α (TNF-α), interleukin-1β (IL-1β), interleukin-6 (IL-6), interleukin-10 (IL-10), interleukin-15 (IL-15), and vascular endothelial growth factor (VEGF) enzyme-linked immunosorbent assay (ELISA) kits were purchased from ZhongShan Golden Bridge Biotechnology Co. (Beijing, China). TNF-α (bs-10802R), IL-1β (bs-6319R), IL-6 (bs-6309R), IL-10 (bs-0698R), IL-15 (bs-1829R), VEGF (bs-1313R), nuclear factor kappa-Bp65 (NF-κBp65) (bs-0465R), transient receptor potential channel-6 (TRPC-6) (bs-2393R), transient receptor potential melastatin-5 (TRPM-5) (bs-9047R) of rabbit polyclonal antibody, and goat anti-rabbit IgG H&L/HRP antibody (bs-40295G-HRP) were purchased from Beijing Biosen Biotechnology Co. (Beijing, China). High-performance liquid chromatography (HPLC)-grade methanol was purchased from American BCR company (Columbus, Ohio, United States). Watsons drinking water (Guangzhou Watsons Food & Beverage Co., Guangzhou, China) was used in this experiment. Other reagents were analytically pure (Tianjin Fuyu Fine Chemical Co., Tianjin, China.).

### Plant Material and Extraction

The plant was collected from Ceheng County, Guizhou, China, in August 2018 and was identified as *Sabia parviflora* Wall. by Professor Qingwen Sun. A voucher specimen (number 20180820) was deposited at the Traditional Chinese Medicine and Ethnic Medicine Laboratory of Guizhou University of Traditional Chinese Medicine.

The air-dried and crude powdered leaves of *S. parviflora* Wall (12.3 kg) were extracted with 70% ethanol (98 L × three times), each time for 2 hours. The combined filtrate was concentrated under reduced pressure to obtain the black extract (2,361.6 g) with a yield of 19.2%. The ethanol extract was extracted five times with petroleum ether, ethyl acetate, and water-saturated n-butanol, respectively. After vacuum drying under reduced pressure, the dried products of the petroleum ether layer (350.5 g), ethyl acetate layer (301.3 g), and water-saturated n-butanol layer (1,295.0 g) were obtained.

### Animals

SPF Sprague–Dawley (SD) rats (180–200 g) were obtained from Changsha Tianqin Biotechnology Co., Ltd (Changsha, China). The standard rat chow was obtained from Shanghai SLAC Laboratory Animal Co., Ltd. (Shanghai, China). The rats were maintained under standard laboratory conditions (temperature 22 ± 1°C, humidity 60 ± 5%, and with 12 h light-dark cycle.) and given standard rodent chow and tap water before initiation of the experiment. All animal experiments were carried out in accordance with the Animal Ethics Committee of Guizhou University of Traditional Chinese Medicine on 25 June 2021 with the Approval No. 20210040.

### Replication of CFA-Induced RA Rat Model and Drug Administration

The rats were adaptively fed for 1 week and their body weight and foot diameter were measured every week thereafter. A total of 96 SD rats were randomly divided into 12 groups of eight animals: 1) normal group, 2) model group, 3) positive control group (dexamethasone), 4) petroleum ether low-dose group, 5) petroleum ether middle-dose group, 6) petroleum ether high-dose group, 7) ethyl acetate low-dose group, 8) ethyl acetate middle-dose group, 9) ethyl acetate high-dose group 10) n-butanol low-dose group, 11) n-butanol middle-dose group, and 12) n-butanol high-dose group. The model was carried out for 2 weeks for all groups, excluding the normal group (each animal was given an intradermal injection of 0.2 ml CFA into the left hind footpad) according to a previously described method (Pan et al., 2017; Bao et al., 2019). The extracts were administered *via* gavage to all groups once a day for 3 weeks, with ordinary feeding after 2 weeks. The dosages of petroleum ether, ethyl acetate, n-butanol (high-, middle-, and low-dose groups), and the dexamethasone positive control groups were 0.64 g/kg, 0.32 g/kg, 0.16 g/kg, and 7.5 mg/kg, respectively.

### Measurement of Body Weight and Changes in Ankle Circumference

The body weight of rats in each experimental group and the normal group were measured and recorded every 7 days from day zero to day 35. The changes in the ankle circumference were also evaluated using a Vernier caliper every 7 days from day zero to day 35.

### Thymus Index and Spleen Index Assay

After collecting blood from the abdominal aorta of rats, the anesthetized rats were killed, and the thymus and spleen were immediately removed and weighed. The index of the thymus and spleen is calculated as follows: thymus index = thymus mass/body mass × 1,000 and spleen index = spleen mass/body mass × 1,000 (Jing et al., 2019).

### Hematoxylin-Eosin Staining Analysis of Synovial Tissue

The rats were killed on day 35, and the ankle joints of rats were cut, fixed in 4% (w/v) paraformaldehyde, and decalcified in 10% ethylene-diamine-tetraacetic acid (EDTA) at 4°C for 1 month. The decalcified ankle tissues were dehydrated, embedded in wax, sectioned, and stained with hematoxylin-eosin. The stained tissues were sealed and then observed and photographed under a microscope using a Ba200 digital micro camera system (magnification, ×100, Motic China Group Co., Ltd., Xiamen, China).

### Immunohistochemical Staining

The expressions of inflammatory proteins in synovial tissue were measured according to the immunohistochemical staining method (He et al., 2018). The treated synovial tissues were incubated with primary antibodies of TNF-α (1:200), IL-1β (1:400), IL-6 (1:400), IL-10 (1:400), IL-15 (1:400), NF-κBp65(1:500), VEGF (1:500), TRPC-6 (1:400), and TRPM-5 (1:400) overnight in a refrigerator at 4°C. The following day, the tissues were washed with phosphate-buffered solution (PBS) three times for 3 min. The tissues were incubated with the anti-goat antibody for 30 min at 37°C and washed with PBS three times for 3 min. The chromogenic agent, 3,3′-diaminobenzidine (DAB), was added for 10 min. The color was observed at room temperature, and the dyeing state was observed under a microscope. Then, the tissues were stained with hematoxylin for 5 min and examined under a microscope using a Ba200 digital micro camera system (magnification, ×100, Motic China Group Co., Ltd., Xiamen, China). The protein expression in synovial tissue was quantitatively analyzed by the average optical density (AOD) of protein according to a previously described method. (Wang, et al., 2021).

### ELISAs for Inflammatory Factors in Serum

The rats were anesthetized by the intraperitoneal injection of 20% urethane, and 4 ml of blood was collected from the abdominal aorta in a labeled red glass. The serum was obtained from blood by cryogenic centrifugation (4°C, 3,000 rpm, and 15 min) and the serum levels of TNF-α, IL-1β, IL-6, IL-10, IL-15, and VEGF were quantified by enzyme-linked immunosorbent assay kits (ELISAs) following the manufacturer instructions.

### HPLC Analysis of the n-Butanol Active Fraction

Chromatographic analysis was performed on an Agilent-1260 HPLC system (Agilent Technologies Inc, Santa Clara, California, United States) coupled with a rapid separation diode array detector (DAD). Chromatographic separation was performed on a YMC-Pack ODS-A column (250 mm × 4.6 mm, 5 μm). The mobile phase consisted of methanol (A) and water (B), and the flow rate was 1.0 ml/min. A linear gradient elution was employed for elution from 35% (A) (v/v) to 100% (A) (v/v) (0–50 min). The DAD detection wavelength was 210 nm, and the column temperature was 25°C. The injection volume was 10 μL.

### Statistical Analysis

Statistical analysis of the experimental data was conducted using GraphPad PRISM version 5.0 software and SPSS 17 software. The results are presented as mean ± standard deviation (SD). The differences between groups were analyzed by one-way analysis of variance (ANOVA) with the *post hoc* test. A *p*-value less than 0.05 was considered statistically significant, and a *p*-value less than 0.01 was considered for significant differences.

## Results

### Effects of *S. parviflora* Wall. Leaf Extracts on Body Weight and Ankle Circumference in Rats

**FIGURE 1:**
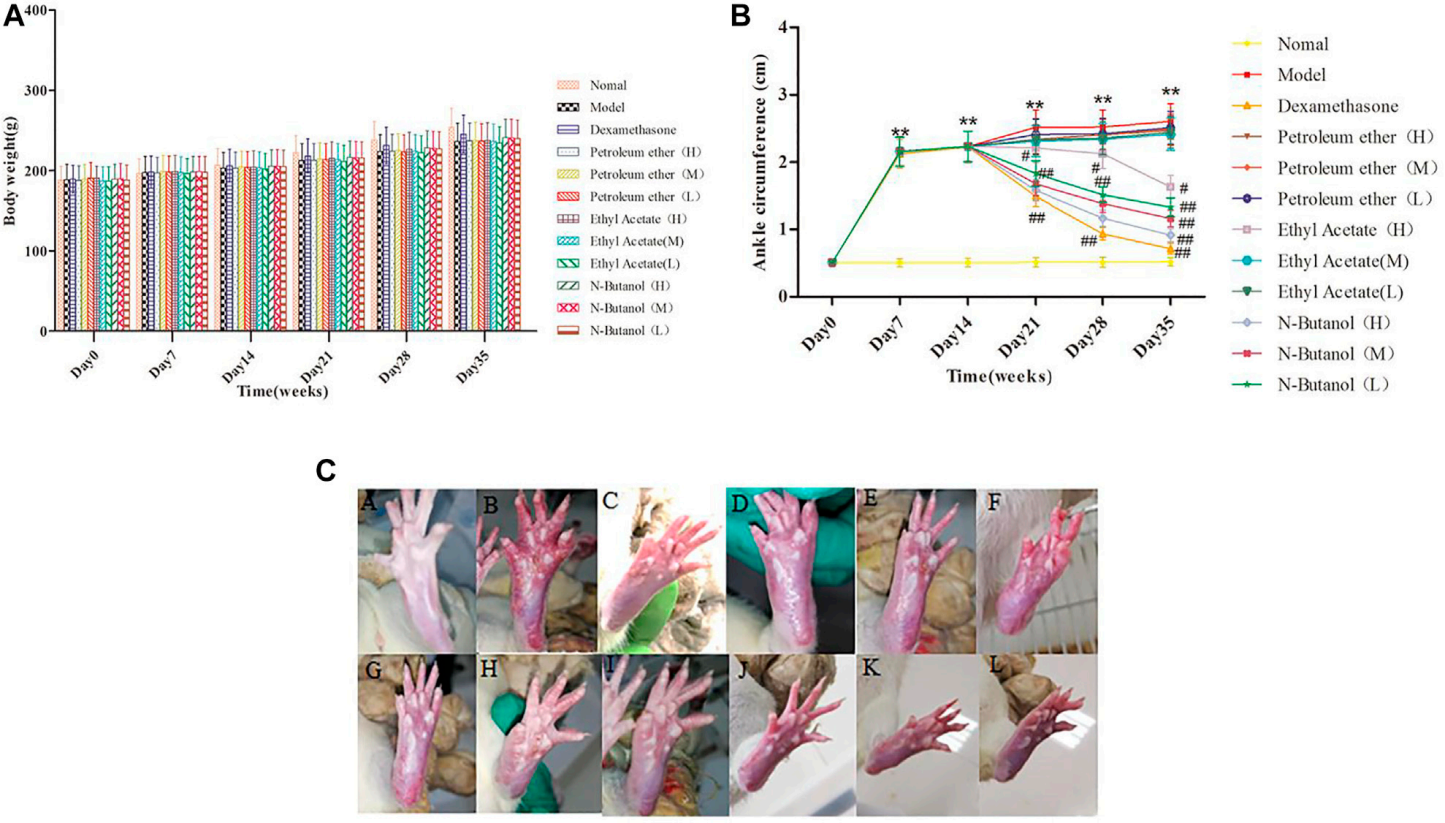
Body weight **(A)** and changes of ankle **(B,C)** in different group of rats. A, B, and C represent normal group, model group, and positive group (dexamethasone), respectively. D, E, and F represent low-dose, middle-dose, and high-dose of fraction of petroleum ether extract, and G, H, and I represent low-dose, middle-dose, and high-dose of fraction of ethyl acetate extract, respectively, and J, K, and L represent low-dose, middle-dose, and high-dose of fraction of n-butanol extract of 70% alcohol-soluble fraction of *S. parviflora* Wall. leaves, respectively. Data are expressed as (mean ± SD), *n* = 8. ^★★^ *p* < 0.01 vs. control group, ^#^ *p* < 0.05 and ^##^ *p* < 0.01 vs. model group.

The body weight of rats in the model group showed a gradual increase, compared to the normal control from day 14 to day 35 (Figure 1A). The administration of *S. parviflora* Wall. leaf extracts*,* only a fraction of n-butanol (0.64 g/kg, 0.32 g/kg, and 0.16 g/kg), and fraction of ethyl acetate (0.64 g/kg) increased body weight from day 21 to day 35, compared to that of the model group (see Supplementary Table S1).

The swelling of the ankle joint of rats from day 7 to day 35 is shown in Figure 1B. The right foot of rats in other groups began to swell on day 7 (*p* < 0.01) compared to the normal rats. Compared with the RA model group, the swelling of rats’ paw of fraction of n-butanol (0.64 g/kg, 0.32 g/kg, and 0.16 g/kg) and fraction of ethyl acetate (0.64 g/kg) of 70% alcohol-soluble fraction of *S. parviflora* Wall. leaf-treated group was alleviated from day 21 (Figure 1B, Figure 1C) (*p* < 0.05 and *p* < 0.01) (see Supplementary Table S2).

### Effects of *S. parviflora* Wall. Leaf Extracts on the Thymus Index and Spleen Index in Rats

**FIGURE 2:**
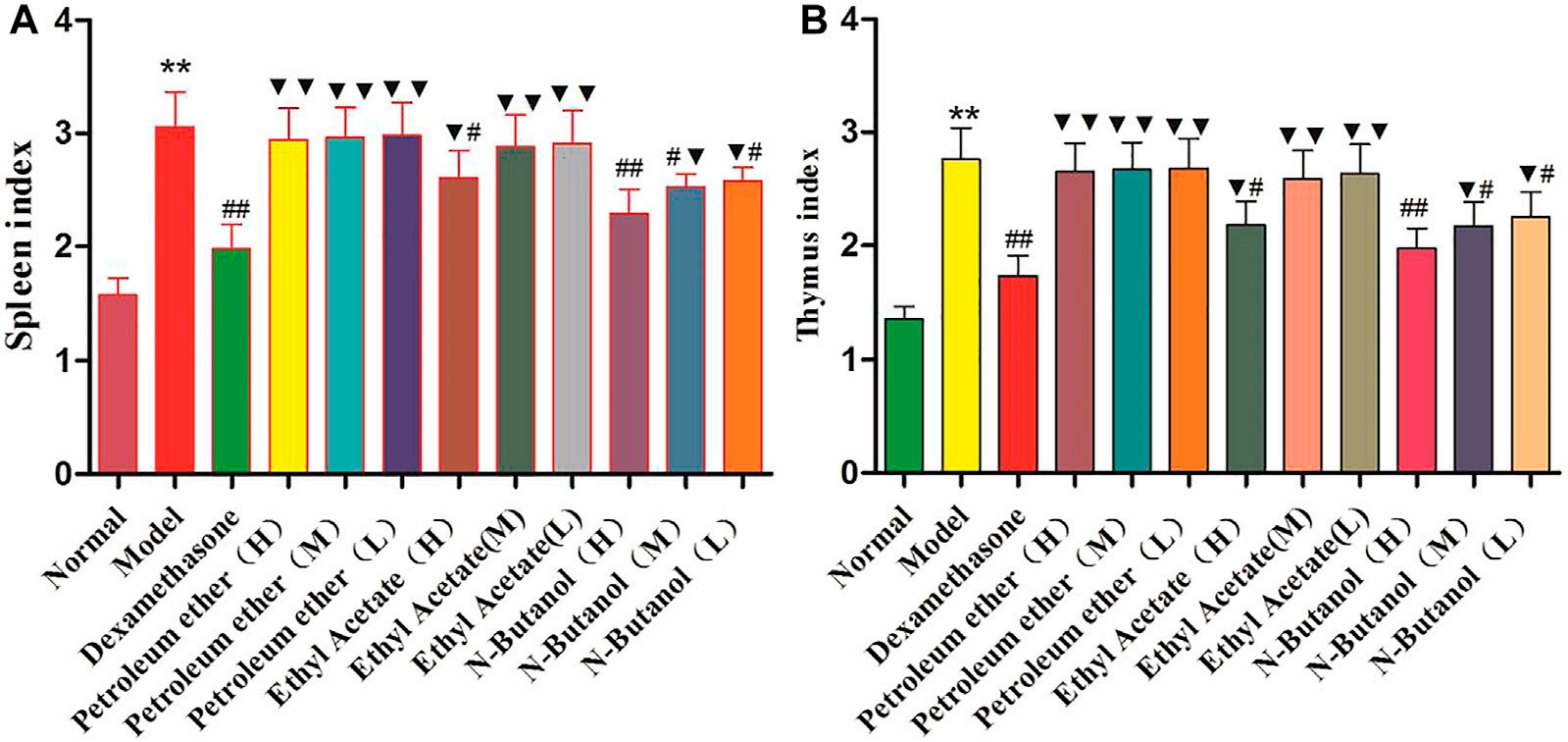
Indexes of the thymus **(A)** and spleen **(B)** in different group of rats. Data are expressed as (mean ± SD), *n* = 8. ^★★^ *p* < 0.01 vs. control group, ^#^ *p* < 0.05, and ^##^ *p* < 0.01 vs. model group, and ^▼^ *p* < 0.05 and ^▼▼^ *p* < 0.01 vs. dexamethasone group.

Compared with the normal group, the thymus index (Figure 2B) and spleen index (Figure 2A) of the RA model group were significantly elevated (*p* < 0.01). In addition, the thymus index and spleen index of the n-butanol fractions (0.64 g/kg, 0.32 g/kg, and 0.16 g/kg) (*p* < 0.05 and *p* < 0.01) and ethyl acetate fraction (0.64 g/kg) (*p* < 0.05) of 70% alcohol-soluble fraction of *S. parviflora* Wall. leaves were lower than those of the model group. Also, the thymus index and spleen index of other treatment groups were higher than those of dexamethasone (*p* < 0.05 and *p* < 0.01) (see Supplementary Table S3).

### Effects of *S. parviflora* Wall. Leaf Extracts on Histopathological Changes in Rat Synovial Tissue

**FIGURE 3:**
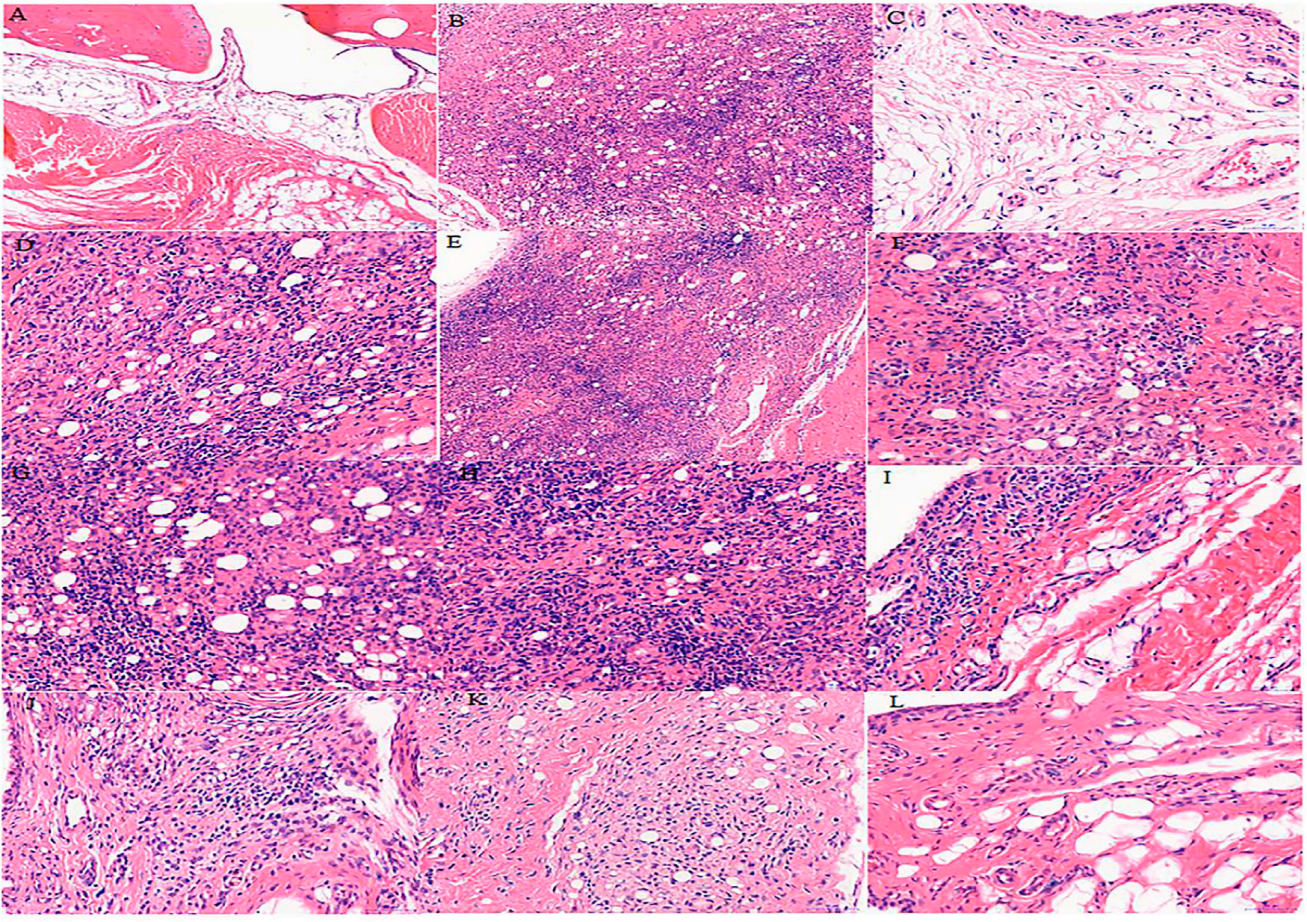
Histological examination of rat synovial tissue in different groups. A, B, and C represent normal group, model group, and positive group (dexamethasone), respectively. D, E, and F represent low-dose, middle-dose, and high-dose of fraction of petroleum ether extract, respectively, G, H, and I represent the low-dose, middle-dose, and high-dose of fraction of ethyl acetate extract, respectively, and J, K, and L represent the low-dose, middle-dose, and high-dose of fraction of n-butanol extract of 70% alcohol-soluble fraction of *S. parviflora* Wall. leaves, respectively.

The cells in the synovial tissue of the normal group were arranged orderly, without inflammatory cell infiltration and vascular proliferation, and the articular cartilage was smooth without damage (Figure 3A). The synovial tissue of the RA model group rats showed obvious synovial hyperplasia, disordered arrangement, and local joint fibrosis. The inflammatory cell infiltration appeared in the synovial stroma and small blood vessels increased in the lower synovial layer, resulting in the formation of a large number of pannus (Figure 3B). Compared with the model group, the n-butanol fractions (0.64 g/kg, 0.32 g/kg, and 0.16 g/kg) and ethyl acetate fraction (0.64 g/kg) of the 70% alcohol-soluble fraction of *S. parviflora* Wall. leaves reduced the pannus in the synovium and local inflammatory cells after treatment for 3 weeks (Figures 3I–L).

### Effects of *S. parviflora* Wall. Leaf Extracts on Inflammatory Cytokine Levels in Rat Serum

**FIGURE 4:**
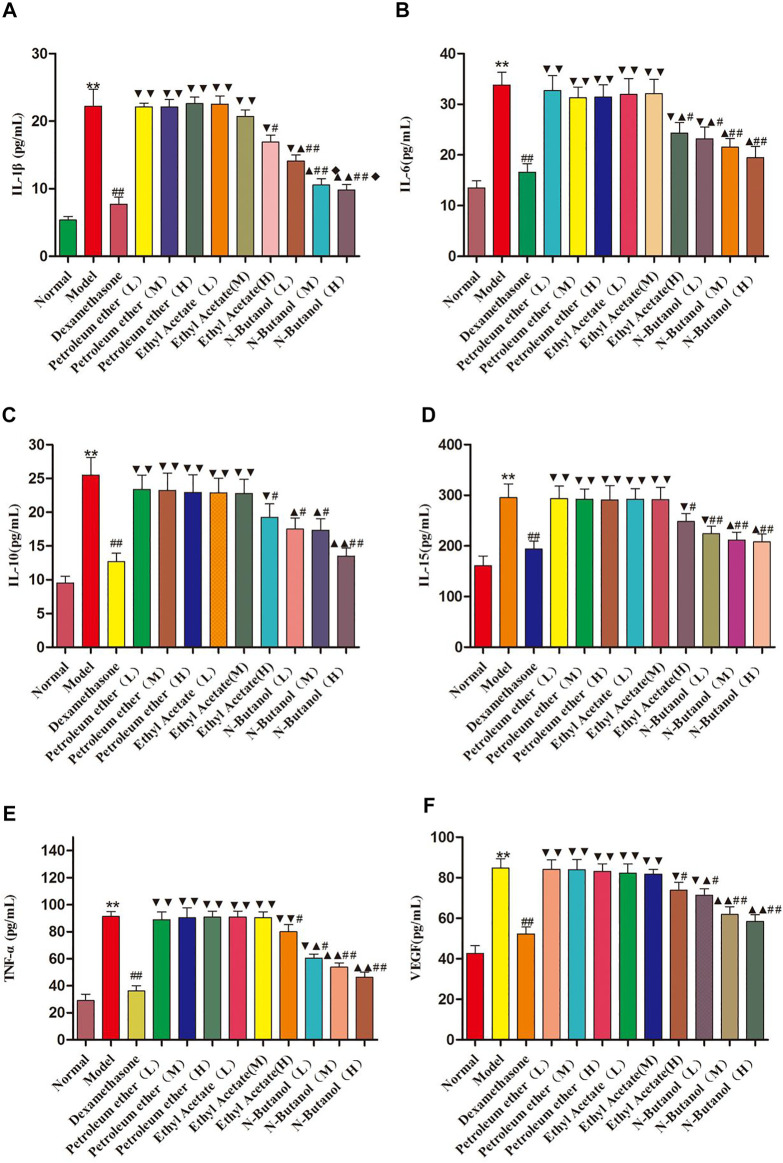
Contents of IL-1β, IL-6, IL-10, IL-15, TNF-α, and VEGF in rat serum of different groups. Data are expressed as (mean ± SD), *n* = 8. ^★★^ *p* < 0.01 vs. control group, ^#^ *p* < 0.05 and ^##^ *p* < 0.01 vs. model group, ^▼^ *p* < 0.05 and ^▼▼^ *p* < 0.01 versus dexamethasone group, ^▲^ *p* < 0.05 and ^▲▲^ *p* < 0.01 vs. different groups of petroleum ether, low-dose, and middle-dose of ethyl acetate group, and ^◆^ *p* < 0.05 versus high-dose of ethyl acetate group.

As demonstrated in Figure 4, the inflammatory cytokine levels of IL-1β, IL-6, IL-10, IL-15, TNF-α, and VEGF in the serum of the model group rats were significantly higher than those in the normal group rats. The IL-1β, IL-6, IL-10, IL-15, TNF-α, and VEGF levels in RA rats treated with the n-butanol fractions (0.64 g/kg, 0.32 g/kg, and 0.16 g/kg) (*p* < 0.05, *p* < 0.01) and ethyl acetate fraction (0.64 g/kg) (*p* < 0.05) of the 70% alcohol-soluble fraction of *S. parviflora* Wall. leaves decreased. In addition, the positive control dexamethasone (7.5 mg/kg/d) also reduced the release of these inflammatory cytokines with significant efficacy (*p* < 0.01). In addition, the inflammatory cytokine levels of other treatment groups were higher than those of dexamethasone (*p* < 0.05 and *p* < 0.01) (see Supplementary Table S4).

### *S. parviflora* Wall. Leaf Fraction of n-Butanol Extract Suppresses the Expression of Inflammatory Proteins in Rat Synovial Tissue

**FIGURE 5:**
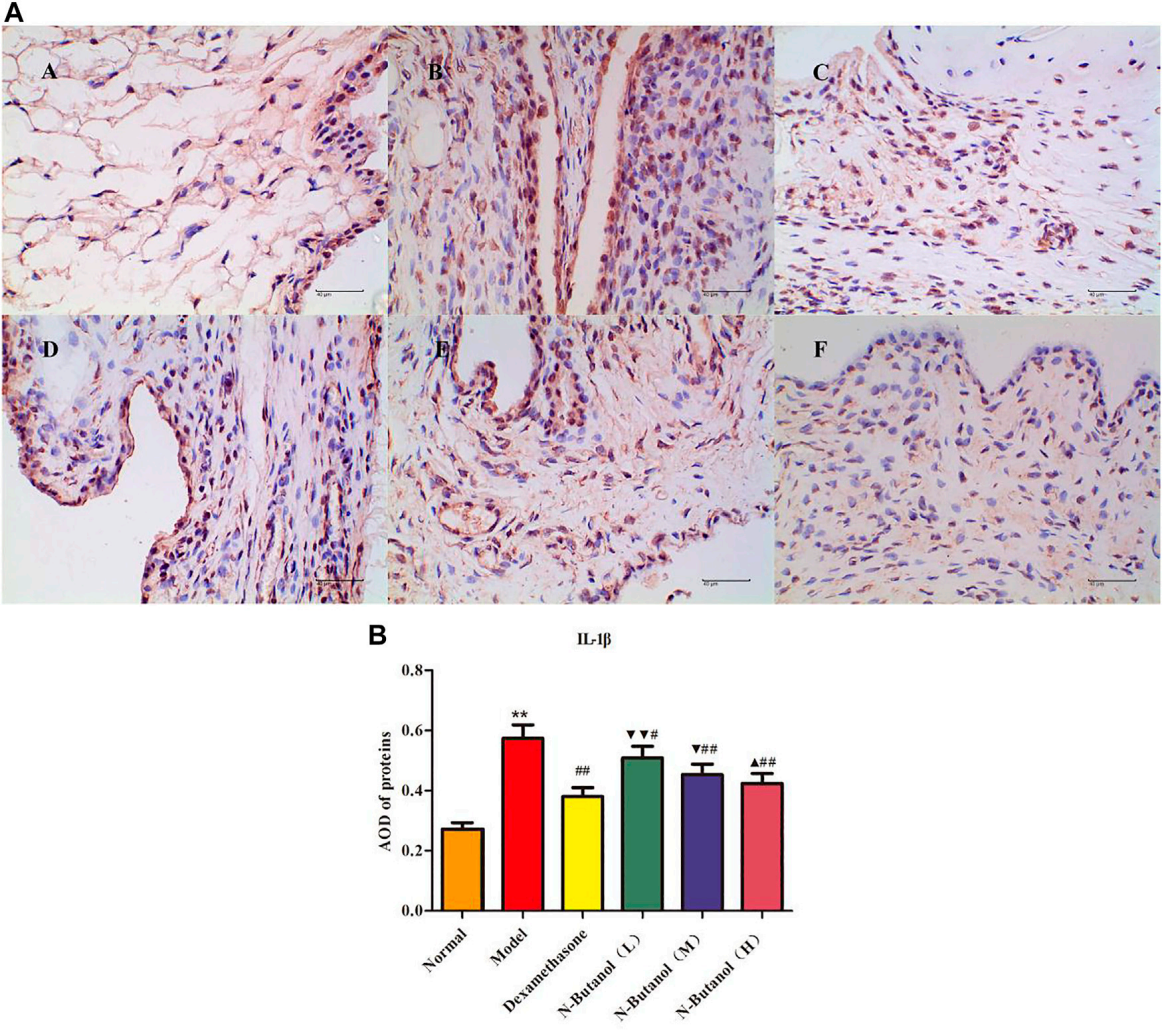
Protein of IL-1β **(A)** expression in rat synovial tissue of different groups and its average optical density (AOD) **(B)**. A, B, and C represent normal group, model group, and positive group (dexamethasone), respectively. D, E, and F represent low-dose, middle-dose, and high-dose of fraction of n-butanol extract of 70% alcohol-soluble fraction of *S. parviflora* Wall. leaves, respectively. Data are expressed as (mean ± SD), *n* = 8. ^★★^ *p* < 0.01 vs. control group, ^#^ *p* < 0.05 and ^##^ *p* < 0.01 vs. model group, ^▼^ *p* < 0.05 and ^▼▼^ *p* < 0.01 vs. dexamethasone group, and ^▲^ *p* < 0.05 vs. low-dose of n-butanol group.

**FIGURE 6:**
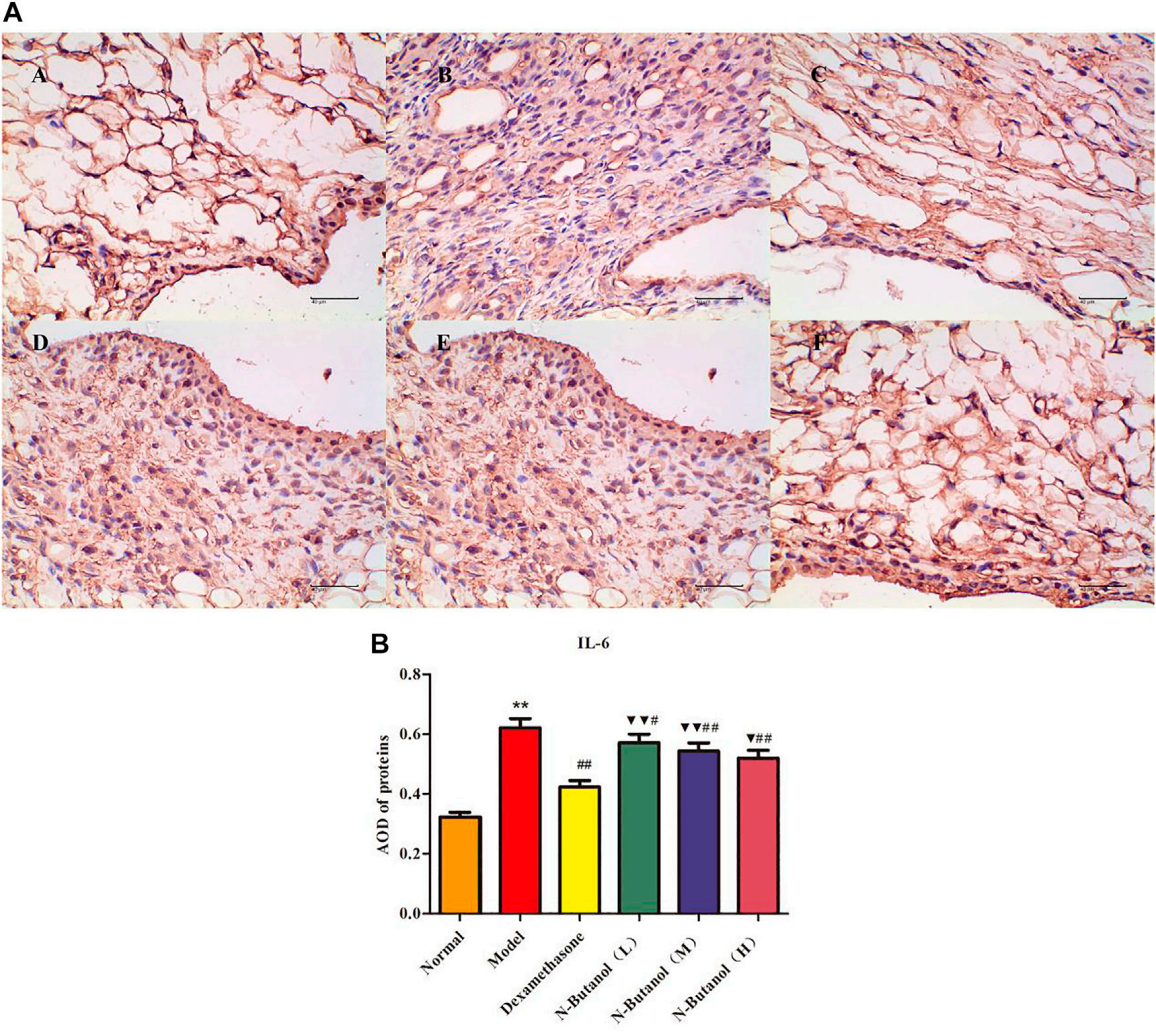
Protein of IL-6 **(A)** expression in rat synovial tissue of different groups and its average optical density (AOD) **(B)**. A, B, and C represent normal group, model group, and positive group (dexamethasone), respectively. D, E, and F represent low-dose, middle-dose, and high-dose of fraction of n-butanol extract of 70% alcohol-soluble fraction of *S. parviflora* Wall. leaves, respectively. Data are expressed as (mean ± SD), *n* = 8. ^★★^ *p* < 0.01 vs. control group, ^#^ *p* < 0.05 and ^##^ *p* < 0.01 vs. model group, ^▼^ *p* < 0.05 and ^▼▼^ *p* < 0.01 vs. dexamethasone group.

**FIGURE 7:**
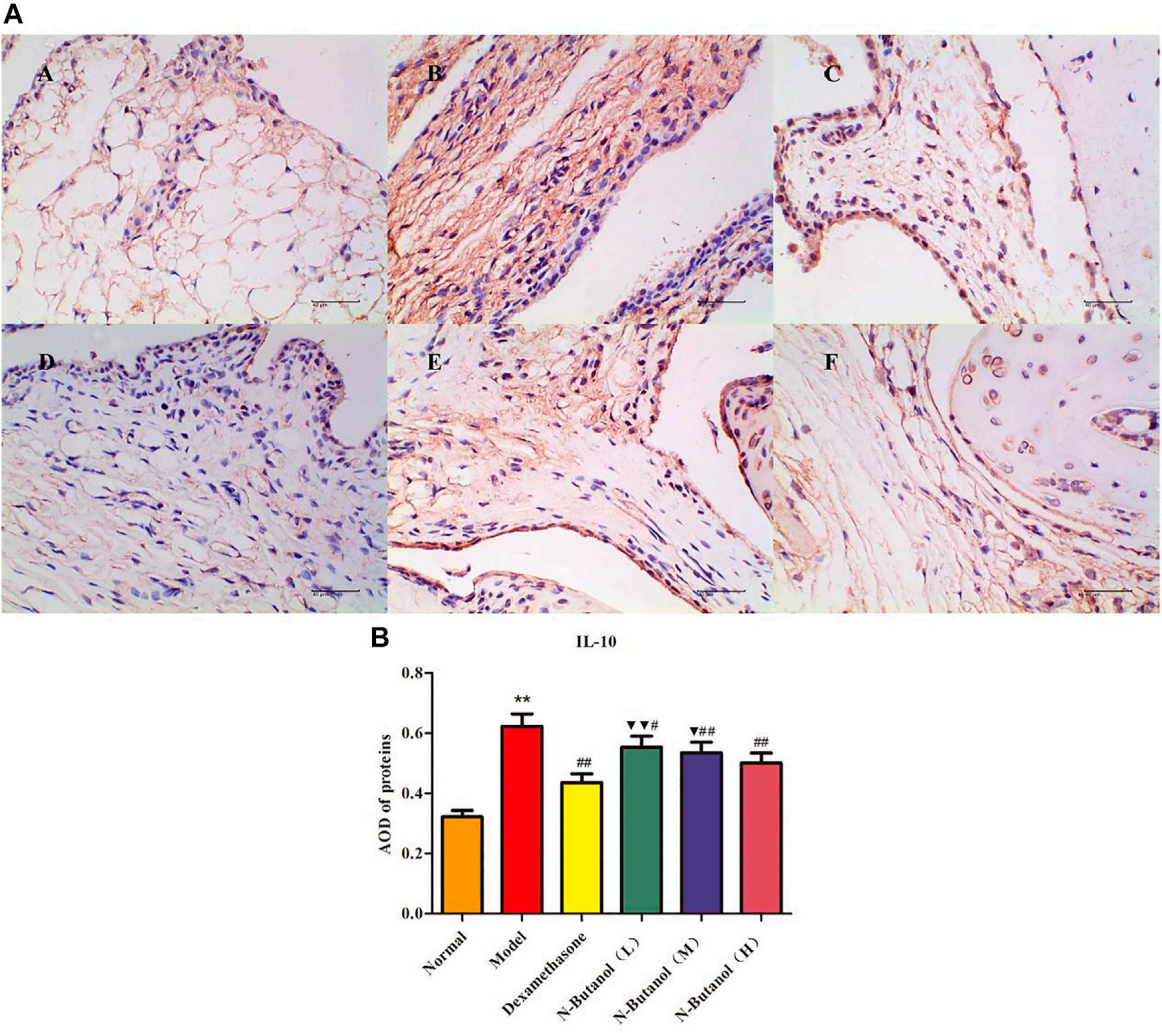
Protein of IL-10 **(A)** expression in rat synovial tissue of different groups and its average optical density (AOD) **(B)**. A, B, and C represent normal group, model group, and positive group (dexamethasone). D, E, and F represent low-dose, middle-dose, and high-dose of fraction of n-butanol extract of 70% alcohol-soluble fraction of *S. parviflora* Wall. leaves. Data are expressed as (mean ± SD), *n* = 8. ^★★^ *p* < 0.01 vs. control group, ^#^ *p* < 0.05 and ^##^ *p* < 0.01 vs. model group, and ^▼^ *p* < 0.05 and ^▼▼^ *p* < 0.01 vs. dexamethasone group.

**FIGURE 8:**
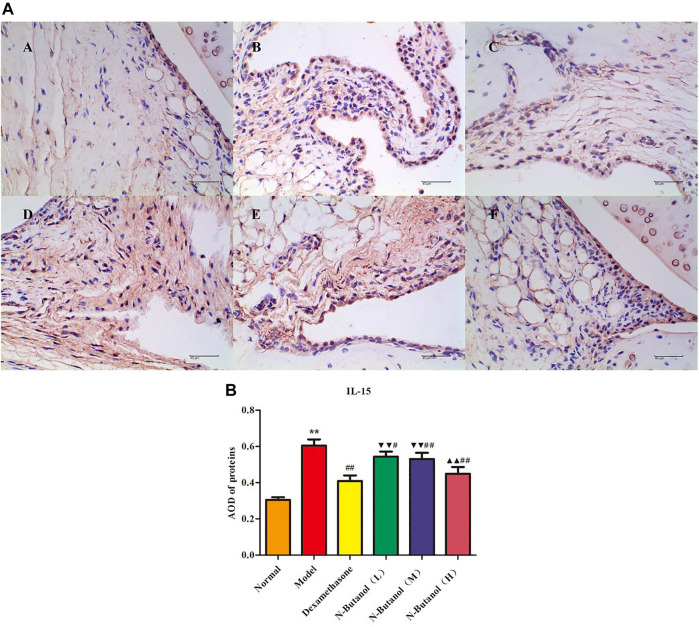
Protein of IL-15 **(A)** expression in rat synovial tissue of different groups and its average optical density (AOD) **(B)**. A, B, and C represent normal group, model group, and positive group (dexamethasone). D, E, and F represent low-dose, middle-dose, and high-dose of fraction of n-butanol extract of 70% alcohol-soluble fraction of *S. parviflora* Wall. leaves, respectively. Data are expressed as (mean ± SD), *n* = 8. ^★★^ *p* < 0.01 vs. control group, ^#^ *p* < 0.05 and ^##^ *p* < 0.01 vs. model group, ^▼▼^ *p* < 0.01 versus dexamethasone group, and ^▲▲^ *p* < 0.01 versus low-dose of n-butanol group.

**FIGURE 9:**
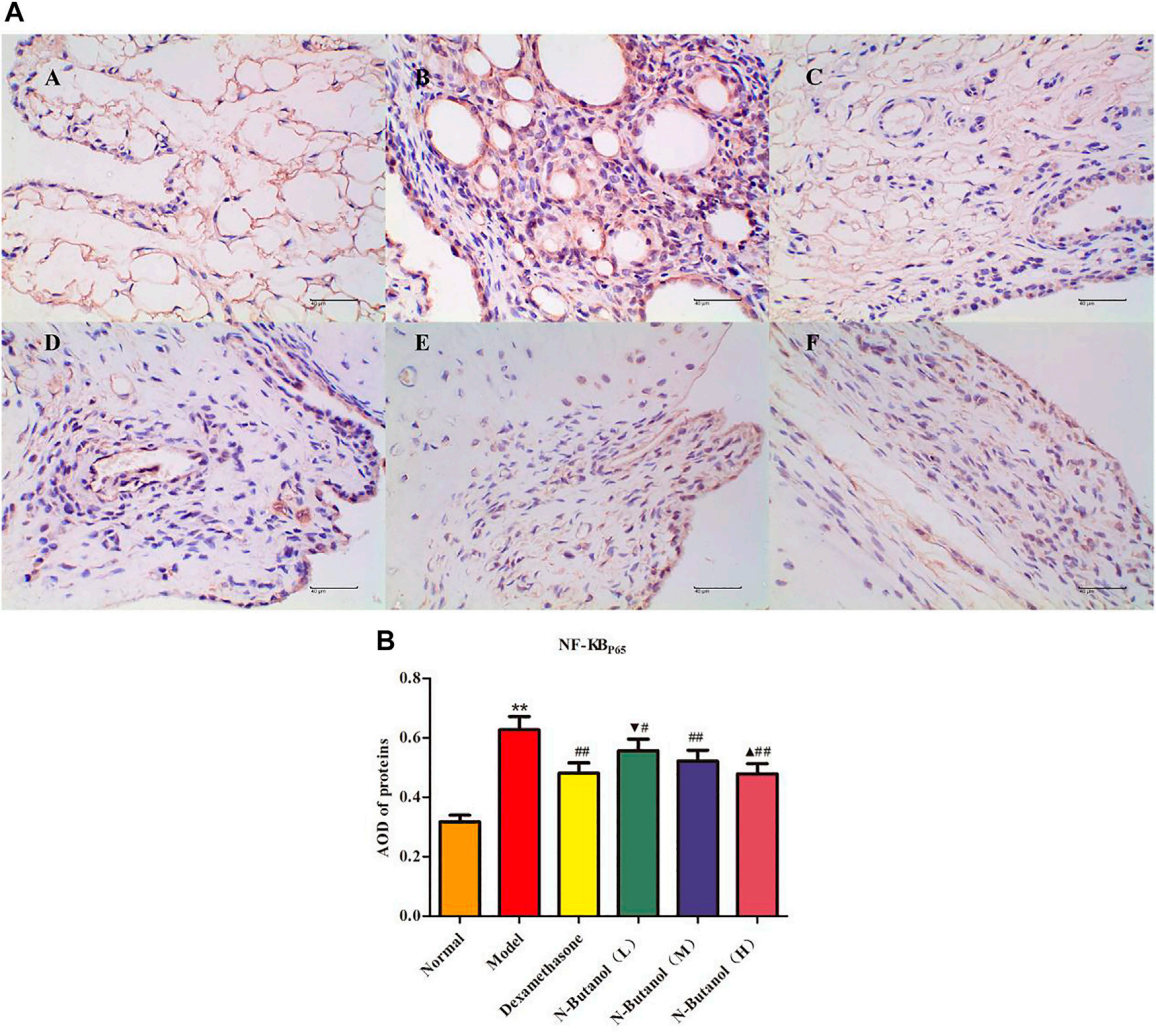
Protein of NF-κBp65 **(A)** expression in rat synovial tissue of different groups and its average optical density (AOD) **(B)**. A, B, and C represent normal group, model group, and positive group (dexamethasone), respectively. D, E, and F represent low-dose, middle-dose, and high-dose of fraction of n-butanol extract of 70% alcohol-soluble fraction of *S. parviflora* Wall*.* leaves, respectively. Data are expressed as (mean ± SD), *n* = 8. ^★★^ *p* < 0.01 vs. control group, ^#^ *p* < 0.05 and ^##^ *p* < 0.01 vs. model group, ^▼^ *p* < 0.05 vs. dexamethasone group, and ^▲^ *p* < 0.05 vs. low-dose of n-butanol group.

**FIGURE 10:**
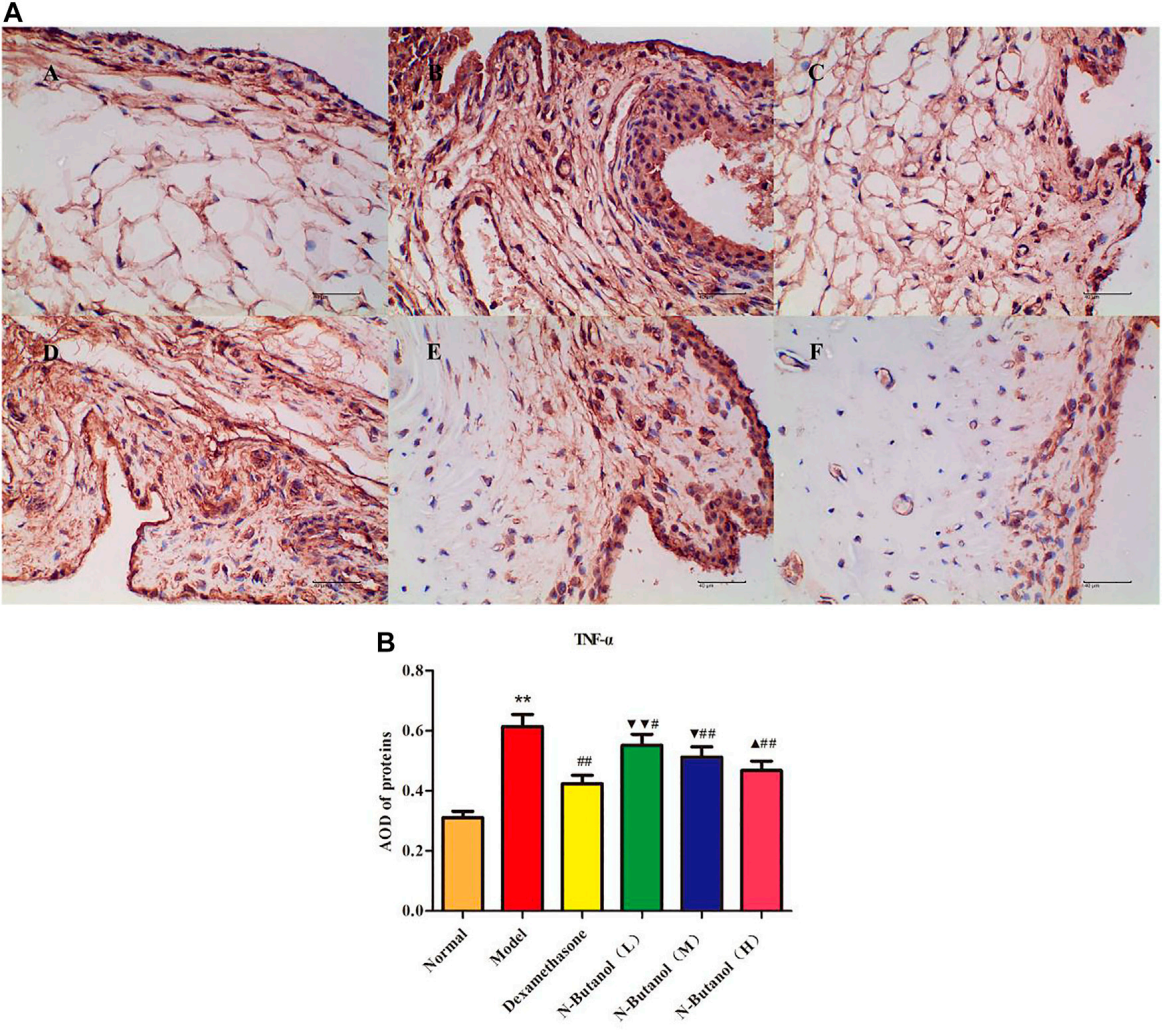
Protein of TNF-α **(A)** expression in rat synovial tissue of different groups and its average optical density (AOD) **(B)**. A, B, and C represent normal group, model group, and positive group (dexamethasone). D, E, and F represent the low-dose, middle-dose, and high-dose of fraction of n-butanol extract of 70% alcohol-soluble fraction of *S. parviflora* Wall*.* leaves. Data are expressed as (mean ± SD), *n* = 8. ^★★^ *p* < 0.01 vs. control group, ^#^ *p* < 0.05 and ^##^ *p* < 0.01 vs. model group, ^▼^ *p* < 0.05 and ^▼▼^ *p* < 0.01 vs. dexamethasone group, and ^▲^ *p* < 0.05 vs. low-dose of n-butanol group.

**FIGURE 11:**
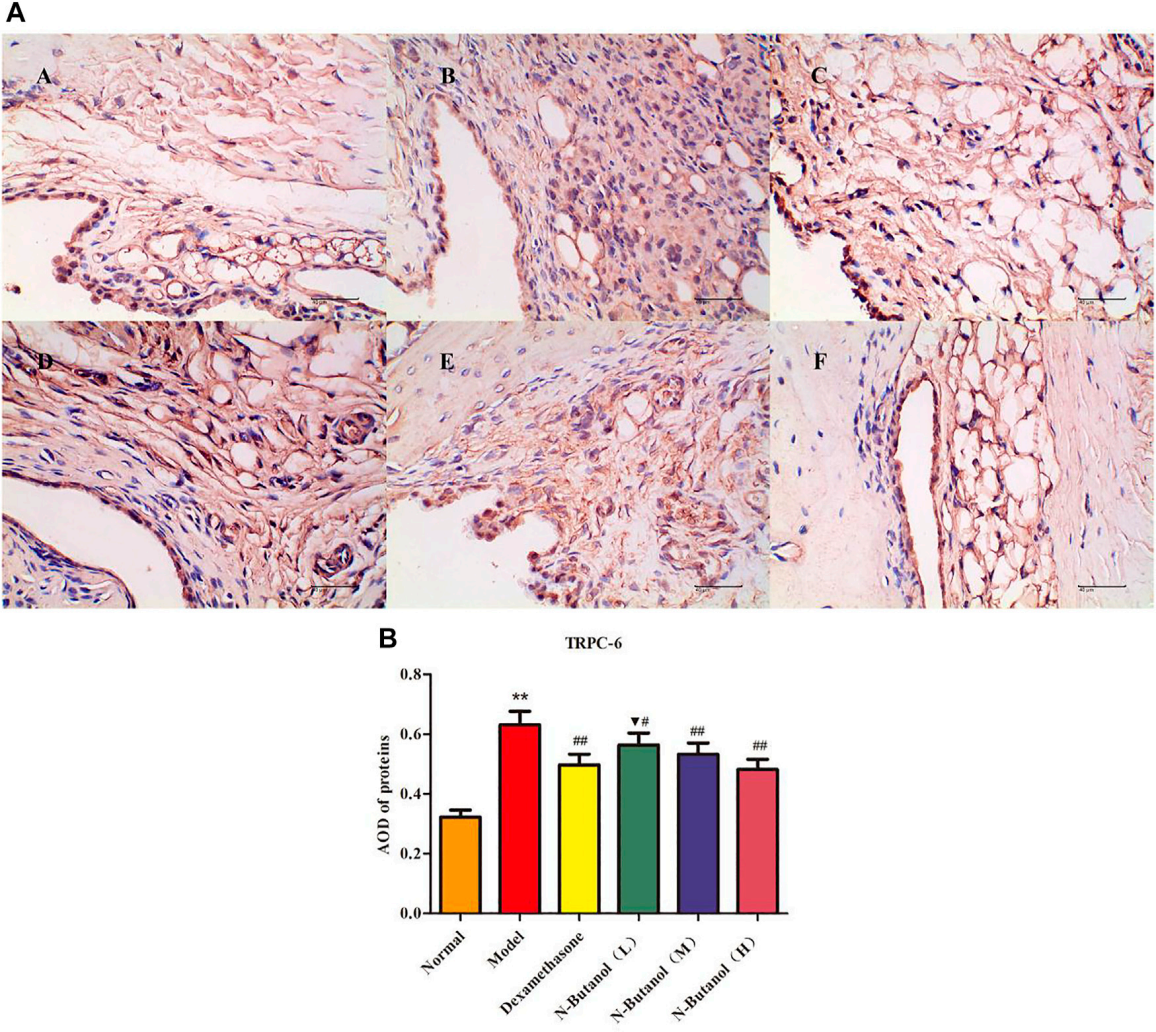
Protein of TRPC-6 **(A)** expression in rat synovial tissue of different groups and its average optical density (AOD) **(B)**. A, B, and C represent normal group, model group, and positive group (dexamethasone). D, E, and F represent low-dose, middle-dose, and high-dose of fraction of n-butanol extract of 70% alcohol-soluble fraction of *S. parviflora* Wall*.* leaves, respectively. Data are expressed as (mean ± SD), *n* = 8. ^★★^ *p* < 0.01 vs. control group, ^#^ *p* < 0.05 and ^##^ *p* < 0.01 vs. model group, and ^▼^ *p* < 0.05 vs. dexamethasone group.

**FIGURE 12:**
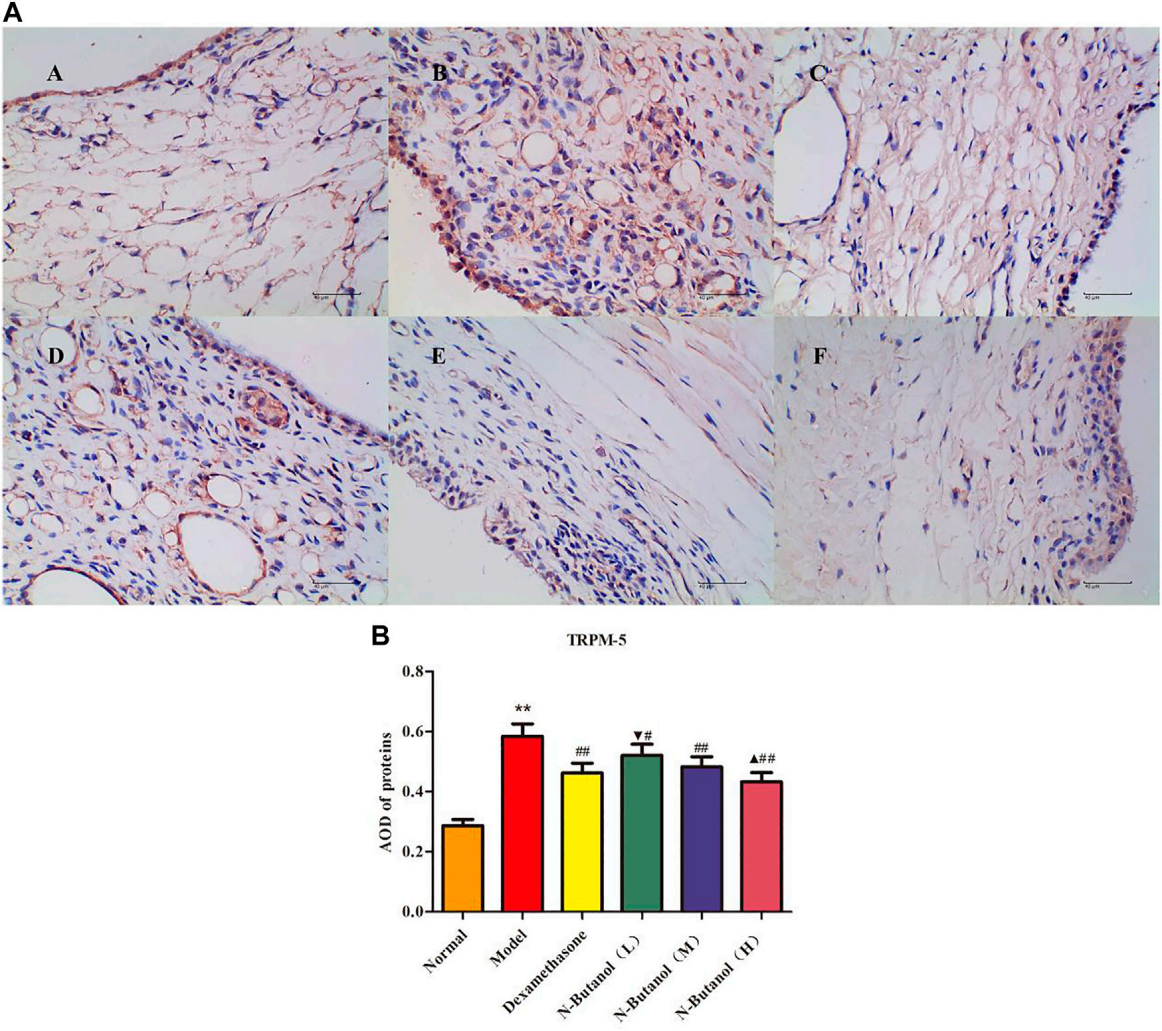
Protein of TRPM-5 **(A)** expression in rat synovial tissue of different groups and its average optical density (AOD) **(B)**. A, B, and C represent normal group, model group, and positive group (dexamethasone). D, E, and F represent low-dose, middle-dose, and high-dose of fraction of n-butanol extract of 70% alcohol-soluble fraction of *S. parviflora* Wall*.* leaves, respectively. Data are expressed as (mean ± SD), *n* = 8. ^&starf;&starf;^ *p* < 0.01 vs. control group, ^#^ *p* < 0.05 and ^##^ *p* < 0.01 vs. model group, ^▼^ *p* < 0.05 vs. dexamethasone group, and ^▲^ *p* < 0.05 vs. low-dose of n-butanol group.

**FIGURE 13:**
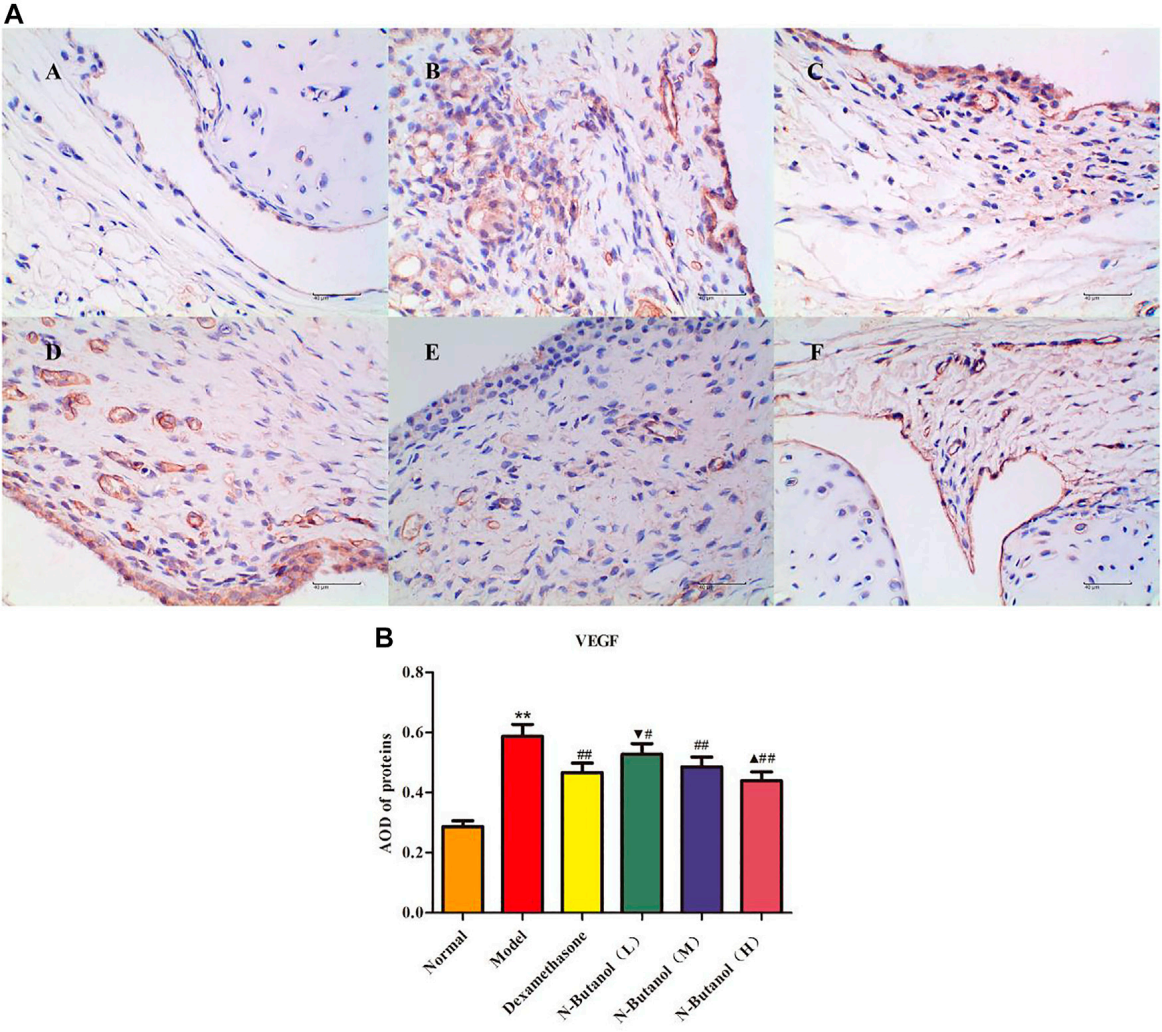
Protein of VEGF **(A)** expression in rat synovial tissue of different groups and its average optical density (AOD) **(B)**. A, B, and C represent normal group, model group, and positive group (dexamethasone). D, E, and F represent low-dose, middle-dose, and high-dose of fraction of n-butanol extract of 70% alcohol-soluble fraction of *S. parviflora* Wall*.* leaves, respectively. Data are expressed as (mean ± SD), *n* = 8. ^&starf;&starf;^ *p* < 0.01 vs. control group, ^#^ *p* < 0.05 and ^##^ *p* < 0.01 vs. model group, ^▼^ *p* < 0.05 vs. dexamethasone group, and ^▲^ *p* < 0.05 vs. low-dose of n-butanol group.

A large number of inflammatory proteins were expressed in RA rat synovial tissue as determined by immunohistochemical examination. Compared with the normal group, the expression of proteins IL-1β, IL-6, IL-10, IL-15, NF-κBp65, TNF-α, TRPC-6, TRPM-5, and VEGF (Figures 5–13) in synovial tissue of RA rats increased significantly (*p* < 0.01). Compared with the RA model group, the expression of these inflammatory proteins in synovial tissue decreased upon treatment with the n-butanol fractions (0.64 g/kg, 0.32 g/kg, and 0.16 g/kg) (*p* < 0.05, *p* < 0.01) of the 70% alcohol-soluble fraction of *S. parviflora* Wall. leaves. Treatment with dexamethasone (7.5 mg/kg/d) also relieved the inflammatory protein expression in synovial tissue of RA rats, with significant efficacy (*p* < 0.01). In addition, the expressions of proteins IL-1β, IL-6, IL-10, IL-15, and TNF-α of other treatment groups were higher than those of dexamethasone (*p* < 0.05) (see Supplementary Table S5 and Supplementary Table S6).

### Identification of Active Components in n-Butanol Active Fraction

**FIGURE 14:**
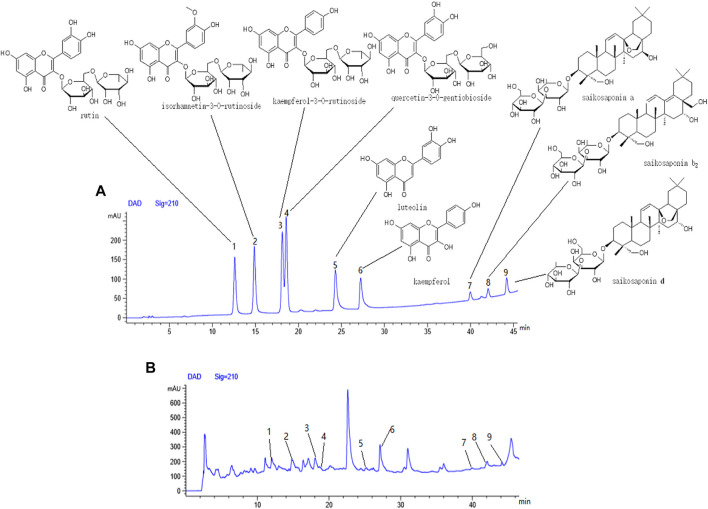
Components from fraction of n-butanol extract of 70% alcohol-soluble fraction of *S. parviflora* Wall. leaves. **(A)** Mixed reference solution, **(B)** sample solution, 1-rutin, 2-isorhamnetin-3-O-rutinoside, 3-kaempferol-3-O-rutinoside, 4-quercetin-3-O-gentiobioside, 5-luteolin, 6-kaempferol, 7-saikosaponin a, 8-saikosaponin b_2_, and 9-saikosaponin d

Six flavonoids: rutin (**1**), isorhamnetin-3-O-rutinoside (**2**), kaempferol-3-O-rutinoside (**3**), quercetin-3-O-gentiobioside (**4**), luteolin (**5**), and kaempferol **6**) and three triterpenoid saponins: saikosaponin a (**7**), saikosaponin b_2_ (**8**), and saikosaponin d **9**) were identified from the n-butanol fraction of the 70% ethanol extract by HPLC (Figure 14A,B).

## Discussion

Rheumatoid arthritis belongs to the category of “arthralgia” (“Bi Zheng” in Chinese) in traditional Chinese medicine, and the main clinical manifestations are pain and numbness in muscles, bones, and joints (Li et al., 2009). Modern medicine shows that the core of RA lesion is the synovial joint, and its main histocytological changes include proliferation of synovial intimal cells, infiltration of inflammatory cells in the subintimal layer of synovium, formation of pannus, which erodes the articular surface, activation of osteoclasts, and mediation of the erosion of mineralized cartilage and soft bone (Schett et al., 2012). Recent studies have validated the crucial roles of osteoprotegerin (OPG)/receptor activator of nuclear factor-κB ligand (RANKL), mitogen-activated protein kinase (MAPK), c-Jun N-terminal kinase (JNK), and Janus kinase (JAK)/signal transducers and activators of transcription (STAT) signal pathways in RA. Among these pathways, many important signal molecules and transcription factors, including NF-κB, c-fos, and c-jun, are involved in the pathogenesis of RA (Gaafar, et al., 2018; Bao, et al., 2019; Kanai, et al., 2020; Zhao et al., 2021; Zhai., et al., 2019). In this study, we have confirmed the antirheumatoid arthritis effects of *S. parviflora* Wall. leaf extracts on RA rats *via* the NF-κB pathway and TRP protein family.

Studies have shown that immune disorders in RA patients are mainly caused by the activation of specific signaling pathways by IL-6, IL-l, TNF-α, and other cytokines. NF-κB is one of the important signal transduction pathways, and it plays a decisive role in regulating inflammatory response (Xia et al., 2018; Zuo et al., 2018). At present, researchers have found that inflammatory cytokines, such as TNF-α and interleukin, in the synovial fluid of RA patients can activate the NF-κB pathway through different ways to cause the RA onset and joint destruction. Targeted blocking of the NF-κB pathway can regulate some cell activities and improve the pathological process of RA (Huang et al., 2019; Pandolfi, et al., 2020). In the present study, the high-dose ethyl acetate fraction, and the n-butanol fractions of the 70% alcohol-soluble fraction of *S. parviflora* Wall. leaves reduced the serum levels of inflammatory factors, IL-1β, IL-6, IL-10, IL-15, and TNF-α in RA rats. These treatment groups also exhibited inhibition of IL-1β, IL-6, IL-10, IL-15, TNF-α, and NF-κB protein expressions in RA rat synovial tissue.

In addition, researchers have found a superfamily of membrane proteins that are found in a wide variety of organisms and participate in sensing external stimuli, namely, TRP. Most subtypes are non-selective Ca^2+^ permeability channels (David., 2003), and most endothelial cell functions depend on intracellular free Ca^2+^ concentration and Ca^2+^ signal transduction. The TRP plays an important role in the regulation of vascular endothelial cell function (Wong et al., 2011). Research on the mechanism of the TRP family, in the regulation of vascular endothelial function, has become a hot spot. Most members of the TRP family have calcium permeability function and are extremely sensitive to exogenous stimuli. Therefore, they have been extensively studied with the aim of revealing the molecular mechanisms of vascular function changes caused by different factors and the occurrence and development of vascular diseases. In particular, TRPC-6 and TRPM-5 proteins are the focus of many studies, as they play key roles in the pathogenesis of vascular diseases (Wu et al., 2020; Shekhar, et al., 2021). In the present work, the n-butanol extract of the 70% alcohol-soluble fraction of *S. parviflora* Wall. leaves could prevent the calcium influx by inhibiting the expression of TRPC-6 and TRPM-5 proteins in RA rat synovial tissue.

Many studies have shown the TRP subfamily members can participate in angiogenesis by affecting the concentration of calcium in endothelial cells. For example, by inhibiting the TRPC-6 channel in human umbilical vein endothelial cells, the cells can stagnate in the G2/M phase and prevent endothelial cell proliferation and lumen formation induced by VEGF, but it has no significant effects on endothelial growth factor (EGF)-induced angiogenesis, indicating TRPC-6 plays an important role in VEGF-induced angiogenesis (Ge et al., 2009). As an important vascular growth factor, VEGF is continuously and highly expressed in the early stage of RA, combined with its receptor through signal transduction. It promotes angiogenesis and increases vascular permeability, resulting in continuous synovitis and pannus formation of RA (Kim et al., 2015; Yamaguchi et al., 2019). Studies have shown that many inflammatory factors directly or indirectly regulate the expression of VEGF in RA patients, and some cytokines such as IL-1, TNF-α, and IL-6 can upregulate the expression of VEGF (Bi et al., 2019; Mumtaz et al., 2020). Therefore, anti-VEGF treatment of RA can delay the progress of the disease, improve clinical symptoms, and provide a new strategy for clinical prevention and treatment of RA. In this work, the n-butanol extract of the 70% alcohol-soluble fraction of *S. parviflora* Wall. leaves inhibited the formation of pannus and reduced the symptoms of synovitis by affecting the protein function of VEGF.

**FIGURE 15:**
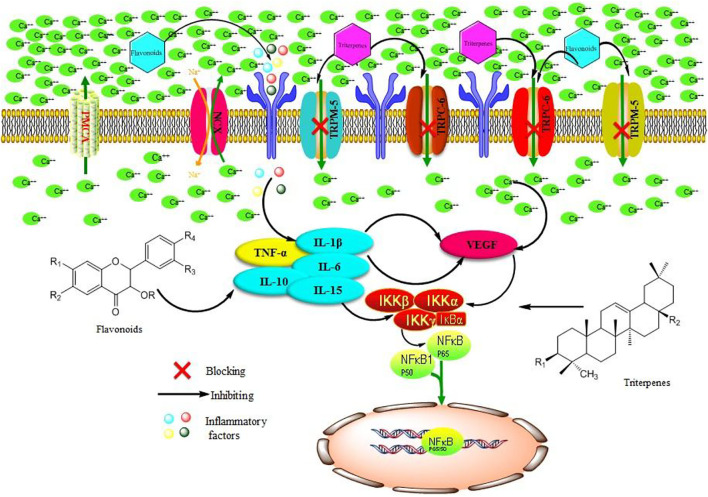
Possible mechanism of n-butanol extract of 70% alcohol-soluble fraction of *S. parviflora* Wall. leaves by acting on the TRP family and NF-κB pathway in the treatment of RA. TNF-α, tumor necrosis factor-α; IL-15, interleukin-15; IL-10, interleukin-10; IL-6, interleukin-6; IL-1β, interleukin-1β; VEGF, vascular endothelial growth factor; TRPM-5, transient receptor potential melastatin-5; TRPC-6, transient receptor potential channel-6; NF-κB, nuclear factor kappa-B; NCX, sodium–calcium exchange; IKKβ, inhibitor of nuclear factor kappa B kinase beta; IKKα, inhibitor of nuclear factor kappa B kinase alpha; IKKγ, inhibitor of nuclear factor kappa B kinase gamma.

At present, alkaloids, flavonoids, and terpenes have been isolated and identified from *S. parviflora* Wall. Among them, pentacyclic triterpenoids are the main type of triterpenoids in *S. parviflora* Wall., and the predominant type of flavonoids is flavonols, which exhibit antiviral and anti-inflammatory activities (Li, et al., 2020; Sun et al., 2021). In this study, we identified five flavonols, one flavonoid, and three triterpenoid saponins from the chromatographic peaks of the n-butanol extract of 70% alcohol-soluble fraction of *S. parviflora* Wall. leaves. These results suggest that the flavonols and triterpenoid saponins in the leaves of *S. parviflora* Wall. may be involved in the treatment of RA. According to chromatographic profile analysis of *S. parviflora* Wall. leaf extracts, we preliminarily summarized and mapped the possible mechanism of *S. parviflora Wall.* leaves in the treatment of RA (Figure 15). The flavonoids and triterpenoids in the effective parts of the leaves of *S. parviflora* Wall. may block the calcium influx by reducing the protein expression of VEGF and inhibiting IL-1β, TNF-α, and other inflammatory factors and protein expression. As a result, the protein expression of the NF-κB pathway is inhibited, playing a role in the treatment of RA.

In this study, we confirmed that the leaves of *S. parviflora* Wall. contain important bioactive compounds that play a role in the treatment of RA by inhibiting the release of inflammatory factors and inhibiting the expression of TRP family protein to reduce the expression of IL-1β, IL-6, TNF-α, and VEGF inflammatory proteins and further inhibit the expression of NF-κB protein in synovial tissue. The combined effects lead to the mitigation of redness and edema in the foot in RA.

## Data Availability

The original contributions presented in the study are included in the article/Supplementary Material; further inquiries can be directed to the corresponding authors.

## References

[B1] Aletaha D. Smolen J. S. (2018). Diagnosis and Management of Rheumatoid Arthritis: a Review. JAMA 320 (13), 1360–1372. 10.1001/jama.2018.13103 30285183

[B2] Bao Y. Sun Y. W. Ji J. Gan L. Zhang C. F. Wang C. Z. (2019). Genkwanin Ameliorates Adjuvant-Induced Arthritis in Rats through Inhibiting JAK/STAT and NF-Κb Signaling Pathways. Phytomedicine 63, 153036. 10.1016/j.phymed.2019.153036 31401534

[B3] Bi W. H. Liu Y. (2019). Research Progress of VEGF in the Occurrence, Development and Treatment of Rheumatoid Arthritis. J. Inn. Mong. Med. Univ. 41 (2), 211–214. 10.16343/j.cnki.issn.2095-512x2019.02.030

[B4] Chatzidionysiou K. Emamikia S. Nam J. Ramiro S. Smolen J. Van Der Heijde D. (2017). Efficacy of Glucocorticoids, Conventional and Targeted Synthetic Disease-Modifying Antirheumatic Drugs: a Systematic Literature Review Informing the 2016 Update of the EULAR Recommendations for the Management of Rheumatoid Arthritis. Ann. Rheum. Dis. 76 (6), 1102–1107. 10.1136/annrheumdis-2016-210711 28356243

[B5] Chen Y. Pan G. Xu W. Sun Q. Wang B. Zhang Y. (2020). Spectrum-effect Relationship Study between HPLC Fingerprints and Antioxidant Activity of Sabia Parvifloraffect Relationship Study between HPLC Fngerprints and Antioxidant Activity of Sabia Parviflora Wall. J. Chromatogr. B Anal. Technol. Biomed. Life Sci. 1140, 121970. 10.1016/j.jchromb.2020.121970 32032821

[B6] Chen Y. Huang T. Yuan C. M. Gu W. Hao X. J. Huang L. J. (2015). Chemical Constituents of *Sabia Parviflora* Wall. Chin. Tradit. Herba1 Drugs. 46 (21), 3146–3150.

[B7] Clapham D. E. (2003). TRP Channels as Cellular Sensors. Nature 426 (6966), 517–524. 10.1038/nature02196 14654832

[B8] Cui R. J. Hu D. Deng L. L. Li J. Mu S. Z. (2022). GC-MS Analysis and Anti-inflammatory Activity of Low Polarity Parts from 3 Species of *Sabia* Genus. Chin. Pharm. 33 (4), 446–451. 10.6039/j.issn.1001-0408.2022.04.11

[B9] Falconer J. Murphy A. N. Young S. P. Clark A. R. Tiziani S. Guma M. (2018). Review: Synovial Cell Metabolism and Chronic Inflammation in Rheumatoid Arthritis. Arthritis Rheumatol. 70 (7), 984–999. 10.1002/art.40504 29579371 PMC6019623

[B10] Fan D. Wang Q. Wang Y. Li Z. Pan L. Yang S. (2018). New Compounds Inhibiting Lipid Accumulation from the Stems of Sabia Parviflora. Fitoterapia 128, 218–223. 10.1016/j.fitote.2018.05.021 29802872

[B11] Gaafar A. G. A. Messiha B. A. S. Abdelkafy A. M. L. (2018). Nicorandil and Theophylline Can Protect Experimental Rats against Complete Freund's Adjuvant-Induced Rheumatoid Arthritis through Modulation of JAK/STAT/RANKL Signaling Pathway. Eur. J. Pharmacol. 822, 177–185. 10.1016/j.ejphar.2018.01.009 29337196

[B12] Ge R. Tai Y. Sun Y. Zhou K. Yang S. Cheng T. (2009). Critical Role of TRPC6 Channels in VEGF-Mediated Angiogenesis. Cancer. Lett. 283 (1), 43–51. 10.1016/j.canlet.2009.03.023 19394138

[B13] He L. Liu C. Sun C. Wang J. Zhi K. Sun D. (2018). Wu-tou Decoction Inhibits Angiogenesis in Experimental Arthritis by Targeting VEGFR2 Signaling Pathway. Rejuvenation Res. 21 (5), 442–455. 10.1089/rej.2017.2011 29385909

[B14] Huang C. C. Chiou C. H. Liu S. C. Hu S. L. Su C. M. Tsai C. H. (2019). Melatonin Attenuates TNF-α and IL-1β Expression in Synovial Fibroblasts and Diminishes Cartilage Degradation: Implications for the Treatment of Rheumatoid Arthritis. J. Pineal. Res. 66 (3), e12560. 10.1111/jpi.12560 30648758

[B15] Jing R. Ban Y. Xu W. Nian H. Guo Y. Geng Y. (2019). Therapeutic Effects of the Total Lignans from *Vitex Negundo* Seeds on Collagen-Induced Arthritis in Rats. Phytomedicine 58, 152825. 10.1016/j.phymed.2019.152825 30831463

[B16] Kanai T. Kondo N. Okada M. Sano H. Okumura G. Kijima Y. (2020). The JNK Pathway Represents a Novel Target in the Treatment of Rheumatoid Arthritis through the Suppression of MMP-3. J. Orthop. Surg. Res. 15 (1), 87–10. 10.1186/s13018-020-01595-9 32131874 PMC7371465

[B17] Kim H. R. Kim K. W. Kim B. M. Cho M. L. Lee S. H. (2015). The Effect of Vascular Endothelial Growth Factor on Osteoclastogenesis in Rheumatoid Arthritis. PloS one 10 (4), e0124909. 10.1371/journal.pone.0124909 25894998 PMC4404365

[B18] Li T.-p. Zhang A.-h. Miao J.-h. Sun H. Yan G.-l. Wu F.-f. (2019). Applications and Potential Mechanisms of Herbal Medicines for Rheumatoid Arthritis Treatment: a Systematic Review. RSC Adv. 9 (45), 26381–26392. 10.1039/C9RA04737A 35685403 PMC9127666

[B19] Li M. S. Ke Y. Q. Guo Z. Y. Liu C. X. Zou K. (2020). Content Determination of Two Triterpenoids in Stems and Leaves of *Sabia Parviflora* Wall. Three. Gorges. Univ. 42 (6), 103–106. 10.13393/j.cnki.issn.1672-948X.2020.06.019

[B20] Li Y. Dai Y. (2009). New Targets in Rheumatoid Arthritis Therapy and the Related Drugs. Prog. Pharm. Sci. 33 (12), 529–536. 10.3969/j.issn.1001-5094.2009.12.001

[B21] Mumtaz M. Hussain N. (2020). Rheumatoid Arthritis and the Role of VEGF Gene: An Overview. J. Sci. Res. Med. Biol. Sci. 1 (2), 75–90. 10.47631/jsrmbs.v1i2.93

[B22] Pala O. Diaz A. Blomberg B. B. Frasca D. (2018). B Lymphocytes in Rheumatoid Arthritis and the Effects of Anti-TNF-α Agents on B Lymphocytes: A Review of the Literature. Clin. Ther. 40 (6), 1034–1045. 10.1016/j.clinthera.2018.04.016 29801753 PMC6792291

[B23] Pan T. Cheng T. F. Jia Y. R. Li P. Li F. (2017). Anti-rheumatoid Arthritis Effects of Traditional Chinese Herb Couple in Adjuvant-Induced Arthritis in Rats. J. Ethnopharmacol. 205, 1–7. 10.1016/j.jep.2017.04.020 28457902

[B24] Pandolfi F. Franza L. Carusi V. Altamura S. Andriollo G. Nucera E. (2020). Interleukin-6 in Rheumatoid Arthritis. Int. J. Mol. Sci. 21 (15), 5238. 10.3390/ijms21155238 PMC7432115 32718086

[B25] Saleem A. Saleem M. Akhtar M. F. Shahzad M. Jahan S. (2020). Moringa Rivae Leaf Extracts Attenuate Complete Freund's Adjuvant-Induced Arthritis in Wistar Rats via Modulation of Inflammatory and Oxidative Stress Biomarkers. Inflammopharmacology 28 (1), 139–151. 10.1007/s10787-019-00596-3 31037575

[B26] Schett G. Gravallese E. (2012). Bone Erosion in Rheumatoid Arthritis: Mechanisms, Diagnosis and Treatment. Nat. Rev. Rheumatol. 8 (11), 656–664. 10.1038/nrrheum.2012.153 23007741 PMC4096779

[B27] Schultz M. Keeling S. O. Katz S. J. Maksymowych W. P. Eurich D. T. Hall J. J. (2017). Clinical Effectiveness and Safety of Leflunomide in Inflammatory Arthritis: a Report from the RAPPORT Database with Supporting Patient Surveyflunomide in Inflammatory Arthritis: a Report from the RAPPORT Database with Supporting Patient Survey. Clin. Rheumatol. 36 (7), 1471–1478. 10.1007/s10067-017-3687-5 28550389

[B28] Shekhar S. Liu Y. Wang S. Zhang H. Fang X. Zhang J. (2021). Novel Mechanistic Insights and Potential Therapeutic Impact of Trpc6 in Neurovascular Coupling and Ischemic Stroke. Int. J. Mol. Sci. 22 (4), 2074. 10.3390/ijms22042074 33669830 PMC7922996

[B29] Siouti E. Andreakos E. (2019). The Many Facets of Macrophages in Rheumatoid Arthritis. Biochem. Pharmacol. 165, 152–169. 10.1016/j.bcp.2019.03.029 30910693

[B30] Sui X. Y. Huang Y. Tan Y. Guo Y. Long C. L. (2011). Molecular Authentication of the Ethnomedicinal Plant Sabia Parviflora and its Adulterants by DNA Barcoding Technique. Planta. Med. 77 (05), 492–496. 10.1055/s-0030-1250468 20979018

[B31] Sun Q. Pan G. Xu W. Lu X. Bai C. Liu M. (2021). Isolation and Structure Elucidation of a New Flavonol Glycoside from Sabia Parviflora. Nat. Prod. Res. 35 (14), 2408–2413. 10.1080/14786419.2019.1679130 31661320

[B32] Wang Y. Liu R. Zhu X. D. (2021). Immunohistochemical Effect of Sishen Pill on PI3K/Akt/mTOR Signal Pathway in Colonic Tissue of Rats with Ulcerative Colitis Model of Spleen Kidney Yang Deficiency. Acta. Lab. Anim. Sci. Sin. 29 (1), 42–48. 10.3969/j.issn.1005-4847.2021.01.006

[B33] Wong C. O. Yao X. (2011). TRP Channels in Vascular Endothelial Cells. Adv. Exp. Med. Biol. 704, 759–780. 10.1007/978-94-007-0265-3_40 21290326

[B34] Wu H. Cui Y. He C. Gao P. Li Q. Zhang H. (2020). Activation of the Bitter Taste Sensor TRPM5 Prevents High Salt-Induced Cardiovascular Dysfunction. Sci. China. Life. Sci. 63 (11), 1665–1677. 10.1007/s11427-019-1649-9 32303962

[B35] Xia Z. B. Meng F. R. Fang Y. X. Wu X. Zhang C. W. Liu Y. (2018). Inhibition of NF-Κb Signaling Pathway Induces Apoptosis and Suppresses Proliferation and Angiogenesis of Human Fibroblast-like Synovial Cells in Rheumatoid Arthritis. Med. Baltim. 97 (23), e10920. 10.1097/MD.0000000000010920 PMC5999456 29879032

[B36] Yamaguchi K. Sudo H. Imai K. (2019). Vascular Endothelial Growth Factor Signaling in VE-Cadherin Expression and Tube-like Formation by Rheumatoid Arthritic Synovial Fibroblast-like Cells. Biochem. Biophys. Res. Commun. 508 (2), 405–409. 10.1016/j.bbrc.2018.11.116 30503342

[B37] Zhai K. F. Duan H. Cui C. Y. Cao Y. Y. Si J. L. Yang H. J. (2019). Liquiritin from Glycyrrhiza Uralensis Attenuating Rheumatoid Arthritis via Reducing Inflammation, Suppressing Angiogenesis, and Inhibiting MAPK Signaling Pathway. J. Agric. Food Chem. 67 (10), 2856–2864. 10.1021/acs.jafc.9b00185 30785275

[B38] Zhao L. J. Wang Y. W. Li Z. F. Fan D. H. Wang Q. Wu B. (2018). Isolation and Identification of Chemical Constituents from *Sabia Parviflora* Wall. Chin. Tradit. Herb. Drugs. 49 (3), 544–548. 10.7501/j.issn.0253-2670.2018.03.006

[B39] Zhao X. Jiang S. Dong Q. Dang J. Liu Z. Han H. (2021). Anti-rheumatoid Arthritis Effects of Iridoid Glucosides from Lamiophlomis Rotata (Benth.) Kudo on Adjuvant-Induced Arthritis in Rats by OPG/RANKL/NF-κB Signaling Pathways. J. Ethnopharmacol. 266, 113402. 10.1016/j.jep.2020.113402 32980481

[B40] Zhou Q. Du W. D. Li Z. F. W Q. Li Y. Feng Y. L. (2022). Two New Compounds from *Sabia Parviflora* Wall. Chin. Tradit. Herb. Drugs. 53 (7), 1939–1942. 10.7501/j.issn.0253-2670.2022.07.002

[B41] Zuo J. Yin Q. Wang Y. W. Li Y. Lu L. M. Xiao Z. G. (2018). Inhibition of NF-Κb Pathway in Fibroblast-like Synoviocytes by α-mangostin Implicated in Protective Effects on Joints in Rats Suffering from Adjuvant-Induced Arthritis. Int. Immunopharmacol. 56, 78–89. 10.1016/j.intimp.2018.01.016 29367090

