# Supplementary material for: Antirheumatoid Arthritic Effects of Sabia parviflora Wall. Leaf Extracts via the NF-κB Pathway and Transient Receptor Potential Protein Family
Source: Front Pharmacol. 2022 Jun 16;13:880350. doi: 10.3389/fphar.2022.880350 (PMC9243545; doi:10.3389/fphar.2022.880350)
Supplement: Supplementary file 1 [file DataSheet1.doc]

**Supplementary Materials**

**Table.1** Body weight in different group of rats (g)

| Groups | Day0 | Day7 | Day14 | Day21 | Day28 | Day35 |
| --- | --- | --- | --- | --- | --- | --- |
| Normal | 188.17±17.23 | 196.62±18.15 | 207.13±20.24 | 222.41±21.34 | 238.35±22.78 | 254.27±23.47 |
| Model | 188.55±19.43 | 197.67±20.12 | 203.11±19.52 | 212.78±20.63 | 223.67±21.13 | 236.45±22.51 |
| Dexamethasone | 189.33±17.36 | 198.15±20.18 | 206.23±20.32 | 218.31±21.13 | 231.45±22.77 | 245.57±23.46 |
| Petroleum ether（H） | 187.87±18.23 | 197.16±19.43 | 203.24±19.77 | 213.18±20.34 | 224.12±21.17 | 237.11±22.25 |
| Petroleum ether（M） | 189.55±18.32 | 199.23±19.78 | 204.78±19.41 | 214.45±20.23 | 224.87±21.24 | 237.43±22.71 |
| Petroleum ether (L) | 190.62±19.41 | 198.18±20.11 | 203.77±20.23 | 213.83±19.76 | 223.65±20.87 | 236.54±21.63 |
| Ethyl Acetate（H） | 190.51±15.32 | 198.87±20.23 | 204.36±20.21 | 215.44±20.37 | 226.51±21.42 | 237.63±22.18 |
| Ethyl Acetate(M) | 187.12±17.80 | 197.34±19.67 | 203.41±20.14 | 213.83±19.55 | 224.15±20.76 | 236.17±21.82 |
| Ethyl Acetate(L) | 186.87±17.68 | 196.43±18.71 | 201.88±19.36 | 211.34±20.13 | 222.13±21.24 | 234.25±20.28 |
| N-Butanol（H） | 189.23±18.91 | 198.14±19.56 | 205.43±20.24 | 216.55±20.21 | 228.26±21.43 | 241.33±22.52 |
| N-Butanol（M） | 189.92±19.11 | 198.55±19.37 | 205.87±20.33 | 216.16±20.11 | 227.87±21.21 | 240.69±23.13 |
| N-Butanol（L） | 188.21±18.56 | 197.88±19.77 | 205.12±20.45 | 215.83±20.15 | 227.14±21.31 | 240.11±22.87 |

Data are expressed as (mean±SD), n = 8.

**Table.2** Ankle circumference in different group of rats (cm)

| Groups | Day0 | Day7 | Day14 | Day21 | Day28 | Day35 |
| --- | --- | --- | --- | --- | --- | --- |
| Normal | 0.508±0.053 | 0.509±0.061 | 0.509±0.064 | 0.511±0.067 | 0.513±0.072 | 0.518±0.061 |
| Model | 0.509±0.051 | 2.159±0.213^★★^ | 2.230±0.232^★★^ | 2.519±0.255^★★^ | 2.523±0.253^★★^ | 2.603±0.261^★★^ |
| Dexamethasone | 0.507±0.052 | 2.123±0.211 | 2.225±0.232 | 1.492±0.155^##^ | 0.936±0.092 ^##^ | 0.715±0.083 ^##^ |
| Petroleum ether（H） | 0.507±0.051 | 2.158±0.218 | 2.229±0.235 | 2.338±0.212 | 2.405±0.233 | 2.471±0.221 |
| Petroleum ether（M） | 0.508±0.053 | 2.161±0.216 | 2.232±0.224 | 2.411±0.233 | 2.422±0.228 | 2.487±0.232 |
| Petroleum ether (L) | 0.508±0.053 | 2.162±0.217 | 2.232±0.233 | 2.413±0.232 | 2.425±0.234 | 2.512±0.242 |
| Ethyl Acetate（H） | 0.508±0.053 | 2.156±0.213 | 2.236±0.228 | 2.212±0.221^#^ | 2.125±0.215^#^ | 1.633±0.171^#^ |
| Ethyl Acetate(M) | 0.509±0.054 | 2.155±0.211 | 2.233±0.232 | 2.312±0.231 | 2.339±0.234 | 2.413±0.241 |
| Ethyl Acetate(L) | 0.507±0.052 | 2.157±0.214 | 2.231±0.227 | 2.332±0.223 | 2.356±0.244 | 2.441±0.235 |
| N-Butanol（H） | 0.508±0.053 | 2.151±0.209 | 2.231±0.234 | 1.583±0.163^##^ | 1.168±0.121^##^ | 0.916±0.097^##^ |
| N-Butanol（M） | 0.507±0.052 | 2.157±0.211 | 2.236±0.211 | 1.678±0.177^##^ | 1.386±0.134^##^ | 1.161±0.123^##^ |
| N-Butanol（L） | 0.509±0.054 | 2.155±0.212 | 2.234±0.225 | 1.833±0.188^##^ | 1.516±0.116^##^ | 1.331±0.142^##^ |

Data are expressed as (mean±SD), n = 8. ^★★^*P* < 0.01 versus control group, ^#^*P* < 0.05 and ^##^*P* < 0.01 versus model group.

**Table.3** The indexes of thymus and spleen in different group of rats

| Groups | The indexes of thymus | The indexes of spleen |
| --- | --- | --- |
| Normal | 1.36±0.11 | 1.58±0.15 |
| Model | 2.76±0.28^★★^ | 3.06±0.31^★★^ |
| Dexamethasone | 1.74±0.17^##^ | 1.98±0.22^##^ |
| Petroleum ether（H） | 2.65±0.25 | 2.94±0.28 |
| Petroleum ether（M） | 2.67±0.24 | 2.96±0.27 |
| Petroleum ether (L) | 2.68±0.26 | 2.98±0.29 |
| Ethyl Acetate（H） | 2.18±0.21^#^ | 2.61±0.24^#^ |
| Ethyl Acetate(M) | 2.59±0.25 | 2.88±0.28 |
| Ethyl Acetate(L) | 2.63±0.26 | 2.91±0.29 |
| N-Butanol（H） | 1.97±0.18^##^ | 2.29±0.22^##^ |
| N-Butanol（M） | 2.17±0.21^#^ | 2.53±0.11^#^ |
| N-Butanol（L） | 2.25±0.22^#^ | 2.58±0.12^#^ |

Data are expressed as (mean±SD), n = 8. ^★★^*P* < 0.01 versus control group, ^#^*P* < 0.05 and ^##^*P* < 0.01 versus model group.

**Table.4** Contents of TNF-α, IL-1β, IL-6, IL-10, IL-15 and VEGF in rats’ serum of different groups (pg/mL)

| Groups | TNF-α | | IL-1β | IL-6 | IL-10 | IL-15 | VEGF |
| --- | --- | --- | --- | --- | --- | --- | --- |
| Normal | | 29.06±4.80 | 5.36±0.52 | 13.51±1.39 | 9.54±0.97 | 160.75±19.38 | 42.63±3.85 |
| Model | | 91.29±3.62^★★^ | 22.21±2.53^★★^ | 33.80±2.51^★★^ | 25.49±2.61^★★^ | 295.77±26.78^★★^ | 84.81±4.53^★★^ |
| Dexamethasone | | 36.15±3.85^##^ | 7.72±1.04^##^ | 16.62±1.65^##^ | 12.68±1.25^##^ | 193.98±15.49^##^ | 52.31±3.43^##^ |
| Petroleum ether（L） | | 88.99±5.75 | 22.15±0.54 | 32.71±2.99 | 23.39±2.13 | 293.41±24.64 | 84.12±4.65 |
| Petroleum ether（M） | | 90.41±7.45 | 22.11±1.11 | 31.33±2.08 | 23.25±2.54 | 292.54±20.05 | 84.07±4.98 |
| Petroleum ether (H) | | 90.76±4.52 | 22.61±0.94 | 31.41±2.43^##^ | 22.93±2.63 | 290.83±28.32 | 83.15±3.70 |
| Ethyl Acetate（L） | | 90.94±4.43 | 22.53±1.22 | 31.93±3.14 | 22.86±2.17 | 292.38±20.83 | 82.32±4.49 |
| Ethyl Acetate(M) | | 90.41±4.43 | 20.70±0.96 | 32.11±2.85 | 22.75±2.10 | 291.90±23.93 | 81.71±2.45 |
| Ethyl Acetate(H) | | 80.17±5.17^#^ | 16.91±1.04^#^ | 24.33±2.10^#^ | 19.23±2.02^#^ | 248.25±16.03^#^ | 73.9±3.85^#^ |
| N-Butanol（L） | | 60.40±3.20^##^ | 14.08±0.92^##^ | 23.18±2.34^#^ | 17.51±1.62^#^ | 224.14±14.59^##^ | 71.36±3.18^#^ |
| N-Butanol（M） | | 53.75±3.14^##^ | 10.56±0.93^##^ | 21.55±1.71^##^ | 17.33±1.73^#^ | 211.84±15.48^##^ | 61.88±3.84^##^ |
| N-Butanol（H） | | 46.30±3.87^##^ | 9.82±0.78^##^ | 19.49±2.19^##^ | 3.51±1.21^##^ | 208.23±15.31^##^ | 58.48±3.35^##^ |

Data are expressed as (mean±SD), n = 8. ^★★^*P*<0.01 versus control group, ^#^*P*<0.05 and ^##^*P*<0.01 versus model group.

**Table.5** Average optical density (AOD) of TNF-α, IL-1β, IL-6, IL-10, and IL-15 proteins expression in rats’ synovial tissue of different groups

| Groups | TNF-α | IL-1β | IL-6 | IL-10 | IL-15 |
| --- | --- | --- | --- | --- | --- |
| Normal | 0.311±0.021 | 0.273±0.021 | 0.323±0.016 | 0.322±0.022 | 0.305±0.015 |
| Model | 0.613±0.041^★★^ | 0.575±0.044^★★^ | 0.622±0.031^★★^ | 0.623±0.041^★★^ | 0.605±0.034^★★^ |
| Dexamethasone | 0.423±0.028^##^ | 0.381±0.029^##^ | 0.424±0.021^##^ | 0.436±0.029^##^ | 0.410±0.031^##^ |
| N-Butanol（L） | 0.552±0.037^#^ | 0.509±0.039^#^ | 0.572±0.029^#^ | 0.553±0.037^#^ | 0.543±0.028^#^ |
| N-Butanol（M） | 0.512±0.034^##^ | 0.453±0.035^##^ | 0.544±0.027^##^ | 0.535±0.036^##^ | 0.531±0.034^##^ |
| N-Butanol（H） | 0.468±0.031^##^ | 0.424±0.033^##^ | 0.520±0.026^##^ | 0.501±0.033^##^ | 0.450±0.037^##^ |

Data are expressed as (mean±SD), n = 8. ^★★^*P*<0.01 versus control group, ^#^*P*<0.05 and ^##^*P*<0.01 versus model group.

**Table.6** AOD of VEGF, NF-κBp65, TRPC6 and TRPM5proteins expression in rats’ synovial tissue of different groups

| Groups | VEGF | NF-κBp65 | TRPC6 | TRPM5 |
| --- | --- | --- | --- | --- |
| Normal | 0.287±0.019 | 0.317±0.023 | 0.323±0.023 | 0.287±0.021 |
| Model | 0.588±0.039^★★^ | 0.627±0.045^★★^ | 0.632±0.045^★★^ | 0.584±0.042^★★^ |
| Dexamethasone | 0.467±0.031^##^ | 0.481±0.034^##^ | 0.498±0.036^##^ | 0.462±0.033^##^ |
| N-Butanol（L） | 0.528±0.035^#^ | 0.556±0.040^#^ | 0.564±0.040^#^ | 0.521±0.037^#^ |
| N-Butanol（M） | 0.486±0.032^##^ | 0.521±0.037^##^ | 0.533±0.038^##^ | 0.482±0.034^##^ |
| N-Butanol（H） | 0.440±0.029^##^ | 0.479±0.034^##^ | 0.482±0.034^##^ | 0.433±0.031^##^ |

Data are expressed as (mean±SD), n = 8. ^★★^*P*<0.01 versus control group, ^#^*P*<0.05 and ^##^*P*<0.01 versus model group.
